# Redox‐Active Microporous Covalent Organic Frameworks for Additive‐Free Supercapacitors

**DOI:** 10.1002/smsc.202400585

**Published:** 2025-03-21

**Authors:** Roman Guntermann, Julian M. Rotter, Apeksha Singh, Dana D. Medina, Thomas Bein

**Affiliations:** ^1^ Department of Chemistry and Center for Nanoscience (CeNS) Ludwig‐Maximilians‐Universität (LMU) Butenandtstraße 11 (E) 81377 Munich Germany

**Keywords:** covalent organic frameworks, ionic liquids, redox‐active, supercapacitors, thin coating

## Abstract

2D covalent organic frameworks (COFs) have garnered significant attention by virtue of their porous nature, structural tunability, and ability to incorporate highly reversible redox‐active groups. These characteristics qualify them for a range of energy storage devices, including supercapacitors, which can assume a pivotal role towards attaining a more sustainable future amid escalating energy needs. Herein, two 2D COFs are reported containing wurster (W) and pyrene (PY) units, WW COF and WPy‐I COF, which demonstrate reversible redox behavior and characteristic pseudocapacitance. Both COFs exhibit high crystallinity demonstrated with X‐ray diffraction analysis, exhibiting a thermal dependence of the intralayer bonding and interlayer stacking arrangement from WPy‐I toward WPy‐II COFs. Additionally, the WW and WPy‐I COFs were grown on glass and stainless‐steel meshes (SSMs) featuring different surface coatings. These coated SSMs proved suitable as current collectors for testing the COFs regarding their specific capacitance, without the need to add any conducting additives, revealing a promising capacitance of 48.9 F g^−1^ for the WW COF. Moreover, these electrodes can be applied in symmetrical supercapacitor devices with an ionic liquid serving as electrolyte. The remarkable performance of the redox‐active Wurster unit identifies it as a promising building motif for COFs with high specific capacitance, even in devices devoid of carbon additives.

## Graphical abstract

A covalent organic framework (COF) and carbonadditive‐free device is introduced for two 2D COFs, WW and WPy‐I, featuring reversible redox behavior and pseudocapacitance. Both COFs exhibit high crystallinity and can be grown on different substrates. They show promising specific capacitance (48.9 F g^−1^ for WW COF) and are successfully used in symmetrical supercapacitors .

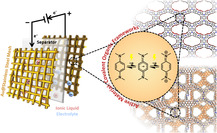

## 1 Introduction

In times of climate change and restrictions on the use of fossil fuels, the integration of renewable energy sources into the electricity grid is of increasing interest. The intermittent nature of renewables requires more flexibility of the power system in order to maintain grid reliability. One promising option is an electrochemical energy storage system using new environmentally friendly and highly efficient materials that can compensate for temporary mismatches between electricity supply and demand.^[^ ^1^, ^2^ ^]^ In this context, the most efficient storage devices and systems working on the principle of electrochemical energy conversion are batteries, fuel cells, and supercapacitors (SCs).^[^ ^3^ ^]^ The latter have emerged as promising alternative to conventional rechargeable batteries due to their high specific capacitance (*C*_s_), high power density, large number of charge–discharge cycles, wide thermal operating range, and low maintenance costs.^[^ ^4^, ^5^ ^]^ Based on their different storage principles, SCs are categorized into electrical double‐layer supercapacitors (EDLSs), pseudocapacitors, and their combination, the so‐called hybrid SCs.^[^ ^6^, ^7^ ^]^ Charges are thereby stored either by non‐Faradaic processes based on the adsorption of electrolyte ions onto the electrode surface for EDLSs or by Faradaic processes resulting from fast surface redox reactions.^[^ ^8^, ^9^ ^]^ Both mechanisms involve intensive interactions between the electrolyte and electrode interfaces. For improved capacitance, excellent thermal stability and a high surface area of the electrode material are required.^[^ ^10^, ^11^ ^]^ The focus is thereby typically on carbonaceous materials such as activated porous carbons or graphene,^[^ ^12^, ^13^, ^14^ ^]^ transition metal oxides,^[^ ^15^ ^]^ and conducting organic polymers.^[^ ^16^, ^17^, ^18^ ^]^ Besides thermal stability and high surface area, the integration of redox‐active sites into organic materials is mandatory for achieving high capacitance. In addition to fulfilling these requirements, crystalline covalent organic frameworks (COFs) offer tunable functionality and have therefore emerged as promising capacitor materials.^[^ ^11^, ^19^ ^]^ The redox properties of the resulting frameworks can be precisely defined by selecting suitable building blocks and positioning them at specific sites in the framework with spatial precision. Considering the subset of 2D COFs constructed from covalently linked 2D polymers that are stacked in the third dimension via dispersive π–π interactions, their long‐range order can promote high electrical in‐plane conductivity through their π‐conjugated intralayer skeletons.^[^ ^20^ ^]^ In some cases, even the π‐stacked molecular columns can lead to pre‐organized conductive pathways for charge carrier transport.^[^ ^21^, ^22^ ^]^ Furthermore, the exceptionally high surface areas of COFs promote efficient ion interactions between electrode and electrolyte. Beyond their application in SCs, the redox‐active characteristics of many COFs have additionally shown significant promise for various types of metal‐ion batteries.^[^ ^23^ ^]^

A highly suitable redox‐active building block motif is the electron‐rich Wurster‐type compound^[^ ^24^ ^]^ containing the molecular unit of *N*,*N*,*N′*,*N′*‐tetraphenyl‐1,4‐phenylenediamine (TPPDA, abbreviated here with “W”). This motif can be easily oxidized and stabilizes radical cations in the π‐system by adopting a symmetric delocalized Robin–Day class III structure.^[^ ^25^ ^]^ TPPDA has recently emerged as a promising building block for diverse functions such as a highly conductive COF for doped materials,^[^ ^26^ ^]^ as basis for bandgap engineering,^[^ ^27^ ^]^ in photocatalytic^[^ ^28^, ^29^, ^30^ ^]^ and electrochromic^[^ ^31^ ^]^ systems or in energy applications.^[^ ^32^, ^33^ ^]^ Using TPPDA‐based COFs combined with carbon further proved promising for electrochemical SCs.^[^ ^34^ ^]^ However, the pure electrochemical charge storage properties of this moiety in COFs without additives have not yet been studied.

Herein, we developed a novel COF, namely, WW COF, based on the condensation of differently functionalized Wurster motifs to take full advantage of the redox‐active building unit in an extended framework. Together with the WPy‐I COF obtained by combining the W motif with a pyrene motif, these two COFs were fully characterized by Fourier‐transform infrared (FTIR) spectroscopy, powder X‐ray diffraction (PXRD), structural simulations, nitrogen sorption, and electron microscopy. Furthermore, the efficient growth of these COFs on precoated stainless‐steel meshes (SSMs) under solvothermal conditions was established, followed by a detailed analysis of their pristine capacitance performance (without additives such as carbon). The application of this electrode preparation method enabled the investigation of the COFs without the additional contribution of carbon to the capacitance, which is frequently ignored in electrochemical studies. Finally, a new symmetrical SC device assembly for pure COFs (without carbon) is introduced, enabling us to investigate the pure capacitance behavior of the corresponding COFs.

## 2 Results and Discussion

### 2.1 Synthesis and Structural Investigations

**Figure 1:**
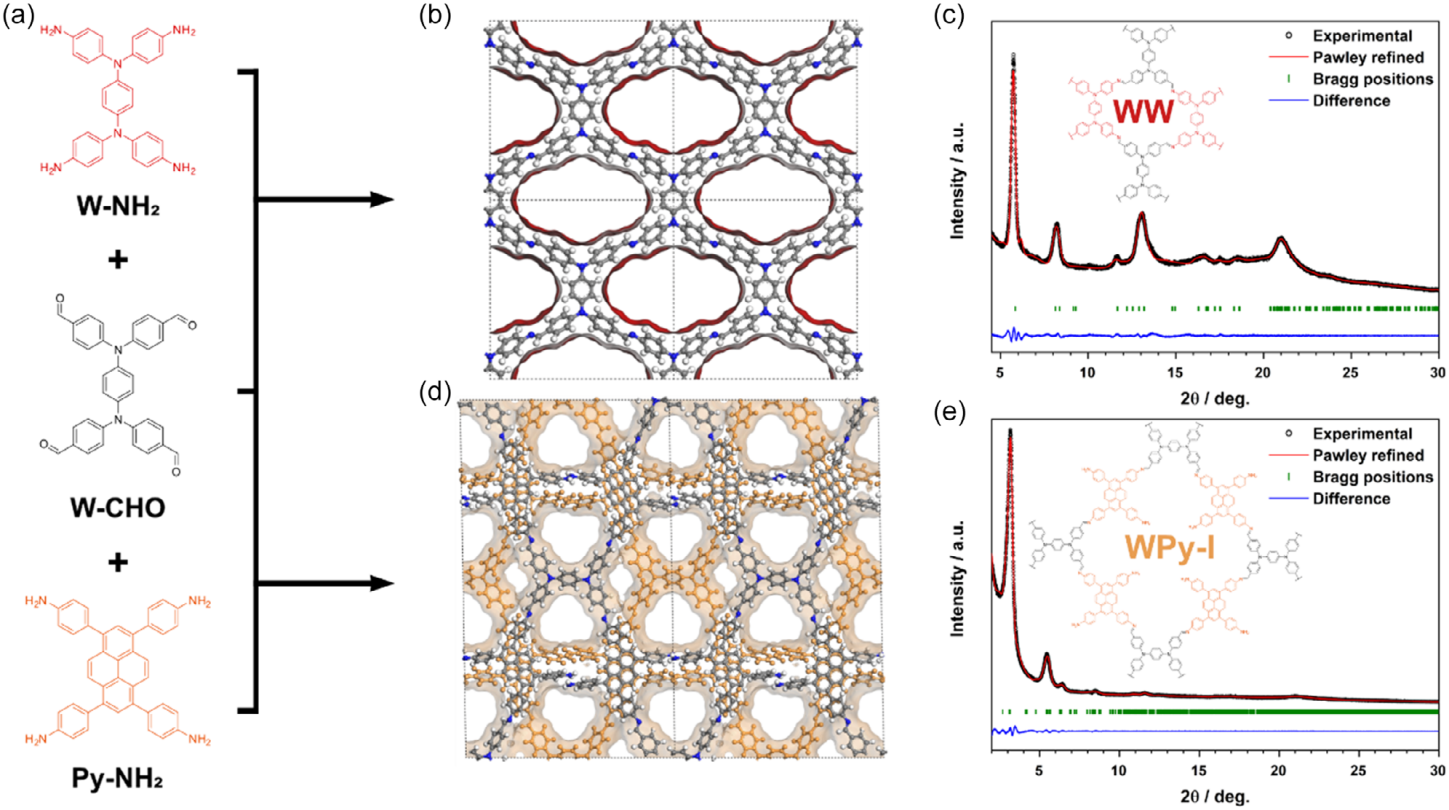
a) Schematic presentation of the synthesis of b) WW and d) WPy‐I COFs via imine condensation with the corresponding structural models (Pawley refined diffraction patterns) of the COFs (b,d). c,e) Experimental PXRD patterns (black dots) of WW and WPy‐I COFs, respectively. The Pawley refinement (red line), the difference plot between the experimental data and the Pawley‐refined PXRD pattern (blue line), and the Bragg positions (indicated by green ticks) are shown. A schematic representation of the corresponding COFs is shown as insets.

The synthesis of the Wurster‐type COFs, bearing redox‐active motifs, occurred through a solvothermal reaction that formed imine bonds via a co‐condensation reaction.^[^ ^35^ ^]^ Specifically, *N*,*N*,*N′*,*N′*‐tetrakis(4‐formylphenyl)‐1,4‐phenylenediamine (W–CHO) and either *N*,*N*,*N′*,*N′*‐tetrakis(4‐aminophenyl)‐1,4‐phenylenediamine (W–NH_2_) or 1,3,6,8‐tetrakis(4‐aminophenyl)pyrene (Py–NH_2_) were utilized to create WW COF or WPy‐I COF, respectively (**Figure** **1**a,b,d). Similar constitutionally isomeric structures^[^ ^27^ ^]^ of the WPy‐I COF were previously reported^[^ ^34^, ^36^, ^37^ ^]^ using the W–NH_2_ as the amine instead of W–CHO and the corresponding aldehyde of the pyrene‐based building block. A solution of mesitylene and benzyl alcohol with a catalytic amount of 6 m acetic acid was chosen as the reaction medium for each building block discussed here. The reaction tubes were sealed and placed in a preheated oven for five days. Subsequent washing steps with tetrahydrofuran (THF) and an extraction with supercritical carbon dioxide (100 bar, 40 °C) serving as solvent were used for purification to prevent pore blocking.^[^ ^38^ ^]^ This process yielded brownish (WW) or light orange (WPy‐I) voluminous and insoluble powders.

The successful imine linkage formation for both COFs was first confirmed by FTIR spectroscopy due to the absence of characteristic stretching bands of the carbonyl (1691 cm^−1^) and amine (around 3350 cm^−1^) functional groups and the formation of a new stretching mode of the C=N bond between 1589 and 1596 cm^−1^ (Figure S1, Supporting Information). PXRD analysis revealed multiple strong and sharp reflections for both samples, indicating the formation of highly crystalline materials (Figure 1c,e). A long shelf life was further observed under ambient conditions, maintaining the desired long‐range order and rendering them robust COFs (Figure S2, Supporting Information).^[^ ^38^ ^]^ Strikingly, for the combination of W–CHO with Py–NH_2_, a crystalline framework is formed within the temperature range of 6–120 °C. Thereby, reactions at low temperatures show a different XRD pattern, indicating a temperature‐dependent structural variation (Figure S3, Supporting Information). To investigate possible reasons for the structural variations, corresponding crystal structures were modeled using the Accelrys Material Studio 7.0 software package and force‐field methods.^[^ ^39^ ^]^ These simulations indicated a preference for a fully connected framework structure, designated WPy‐II, at elevated temperatures (Figure S4, Supporting Information). Furthermore, the spatial configuration of the building blocks within this structure was investigated by simulating structures with the longer axis of the W motif being aligned horizontally, vertically or alternately against the longer axis of the pyrene motif, each time in an eclipsed or staggered layered structural model (Figure S5–S7, Supporting Information). This analysis indicated that an alternating configuration of Wurster units was the most probable fully connected structure. However, in this study we focus on the kinetically favored COF WPy‐I obtained at lower temperatures with the highest consistent crystallinity achieved at 50 °C. Based on previous reports on similar COFs composed of tetratopic building blocks exhibiting frustrated bonded networks,^[^ ^37^, ^40^ ^]^ we focused our structural analysis on frameworks with unreacted amine or aldehyde linkages. The simulations of frustrated frameworks revealed a matching XRD pattern for a WPy structure with pyrene serving as linear connection between the Wurster moieties in a staggered stacking topology (Figure 1d,e, S8, and S9, Supporting Information). Pawley refinement of the simulated structure resulted in good agreement between the experimental and simulated pattern of the WPy‐I COF. Accordingly, the reflections at 3.18°, 5.45°, and 6.42° 2*θ* were attributed to *hkl* (020), (200), and (220) in the space group *P1*, respectively.

For the WW COF, only a fully bonded framework was obtained, with the highest crystallinity reached at 100 °C. The fully bonded framework formation was confirmed by the simulated PXRD data of an eclipsed stacking topology that agrees well with the corresponding experimental results (Figure 1c and S10, Supporting Information). The simulated unit cell is constructed of building blocks with the same core structure but with different reactive groups necessary for the condensation reaction forming the imine bonds. The diffraction reflections of the WW‐COF were indexed on a pseudotetragonal unit cell in the *P2/m* space group as follows: (110) at 5.72°, (200)/(020) at 8.19°, (220) at 11.63°, (130) at 13.07°, and (001) at 20.99° 2*θ*. The observed (001) reflection can be assigned to the π–π‐stacking distance of the 2D COF layers with a *d*‐spacing value of 4.29 Å.

The COFs’ porosity was evaluated via nitrogen physisorption measurements at 77 K. The isotherms exhibit an IUPAC Type I shape with a high nitrogen uptake at low partial pressure, which typifies microporous materials (Figure S11a,c, Supporting Information). Utilizing the Brunauer–Emmett–Teller (BET) method, we calculated a surface area of 493 m^2^ g^−1^ for the WW COF and of 1181 m^2^ g^−1^ for the WPy‐I COF, along with pore volumes for these COFs of 0.279 and 0.501 cm^3^ g^−1^ at *p/p*_*0*_ = 0.2, respectively. The experimental (nominal) BET surface area of microporous materials may not accurately reflect the actual surface area due to the limited multilayer adsorption caused by small pore widths, rendering the BET model less valid in this case.^[^ ^41^ ^]^ The experimental pore size distribution, determined via the quenched solid density functional theory method for 1D cylindrical pores and carbon surfaces, indicated a pore size of 1.55 nm for the WW–COF, consistent with the simulated value of ≈1.3 nm (Figure S11b, Supporting Information). Sorption analysis of the WPy‐I COF revealed one pore size of ≈0.85 nm and further indicates an even smaller one below 0.60 nm (Figure S11d, Supporting Information). These results provide strong additional support for the predicted structure of a frustrated framework in a staggered stacking topology, with simulated pore sizes of 0.99 and 0.56 nm. A thermal stability of up to 330 °C (WPy‐I COF) and up to 400 °C (WW COF) under synthetic air flow and dynamic heating was further observed using thermogravimetric analysis (Figure S12, Supporting Information).

**Figure 2:**
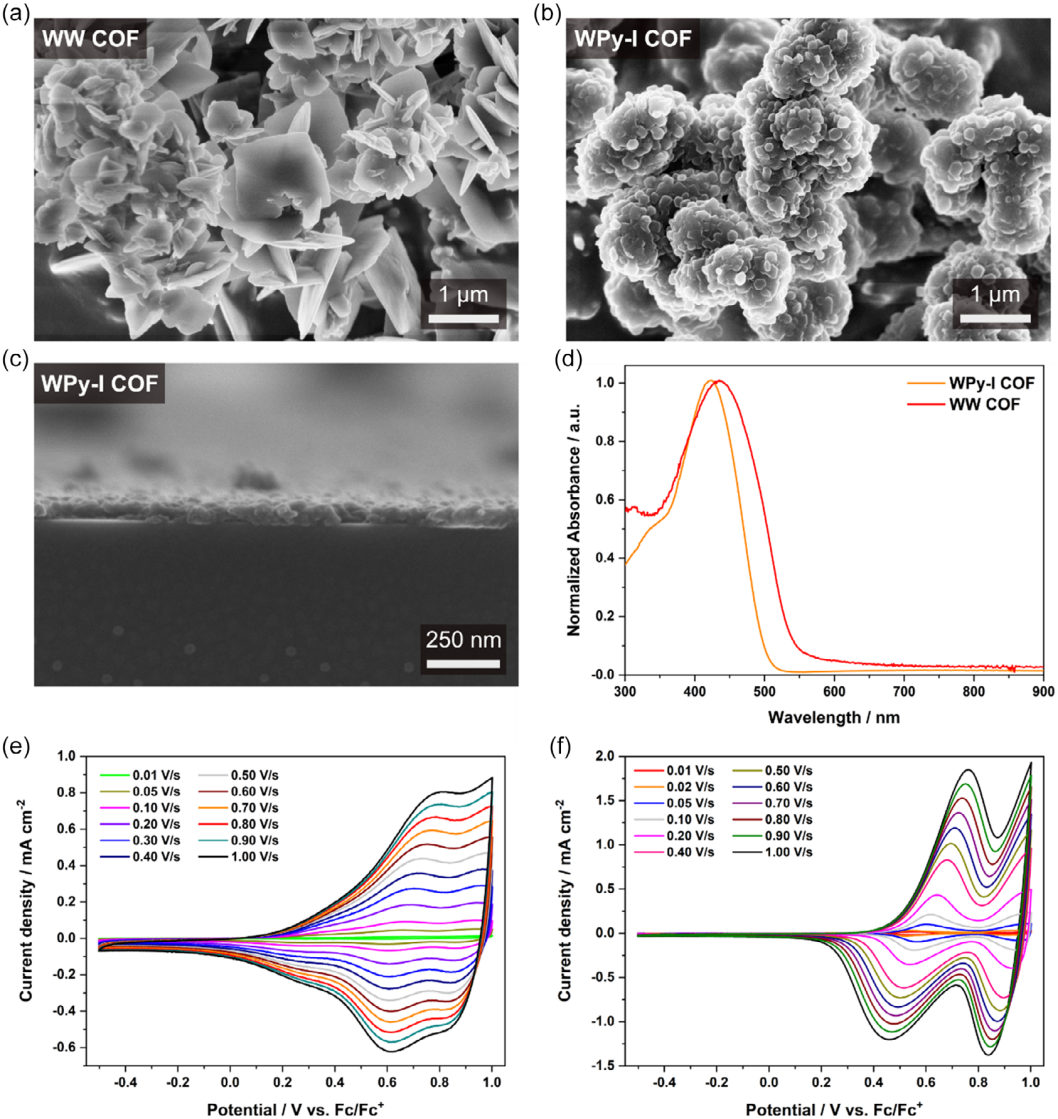
SEM images of a) WW COF revealing intergrown plate‐like particles and b) the WPy‐I COF displaying agglomerated spherical particles. c) Cross‐sectional SEM image of WPy‐I COF thin film on glass. d) Absorption spectra of WW COF (red) and WPy‐I COF (orange) films with absorption maxima at 435 and 424 nm, respectively. Cyclic voltammograms of e) WW and f) WPy‐I COF films on ITO substrates at different scan speeds using an acetonitrile solution containing tetrabutylammonium hexafluorophosphate (TBA PF_6_) as electrolyte.

Next, the bulk COF materials were analyzed by scanning electron microscopy (SEM), revealing well‐defined crystal morphologies for both frameworks. The WW COF powder displayed a fairly uniform morphology of intergrown plate‐like particles with a size of ≈400 nm and an individual particle thickness of around 40 nm (**Figure** **2**a and S13, Supporting Information). The bulk morphology of the WPy‐I COF comprises spherical particle shapes agglomerating along nanofibers (Figure 2b). Perpendicular to the surface of the individual spherical agglomerates, predominantly small hexagonal particles protrude with an average size of 110 nm (Figure S13, Supporting Information). These densely intergrown hexagonal particle shapes were further confirmed by transmission electron microscopy (TEM) (Figure S16, Supporting Information).

The bulk COF materials were analyzed further as pressed pellets to determine their electrical conductivities (in undoped form) at room temperature using a van der Pauw four‐probe setup (Table S1, Supporting Information). The resulting conductivity values for the WW and WPy‐I COFs were found to be at the order of 10^−8^ S cm^−1^, which is within a range similar to other Wurster‐type COFs.^[^ ^26^ ^]^

### 2.2 COF Films

To examine optical and electrical properties of the COFs, we grew COF films on glass and indium tin oxide (ITO) substrates under solvothermal conditions. For the film synthesis, we applied the standard procedure for powder synthesis on a larger scale while additionally placing a substrate horizontally in the COF reaction vessel.^[^ ^42^, ^43^ ^]^ SEM top‐view images of the brownish WW COF film reveal a uniform coverage with primarily plate‐like particles adhering randomly to the surface (Figure S14, Supporting Information), with a thickness of around 2 μm shown by cross‐section. The yellow WPy‐I COF film displayed a more closely packed thin layer of intergrown particles measuring ≈80 nm in thickness (Figure 2c and S15, Supporting Information). Grazing incidence wide‐angle X‐ray scattering analysis evidenced no preferred orientation of both COF films, as already indicated by the random orientation of the WW COF particles in the SEM images (Figure S17, Supporting Information).

Next, the COF films were analyzed using UV–vis spectroscopy (Figure 2d). The resulting spectra exhibit a broad absorption ranging from 300 to 500 or 550 nm, with the WPy‐I COF and WW COF achieving a maximum absorbance intensity at 424 and 435 nm, respectively. The WW COF absorbs light deeper into the visible spectrum, as reflected by the film's brownish hue compared to the WPy‐I COF's distinct yellowish color. Optical bandgap values of 2.27 eV (WW COF) and 2.46 eV (WPy‐I COF) were determined by generating Tauc plots from the UV–vis spectra for a direct bandgap transition model (Figure S18, Supporting Information).

The tertiary triphenylamine groups in the two COFs can undergo reversible redox processes, rendering them suitable for studying electrochemical phenomena that may be applicable for energy storage applications (e.g., SCs). To first examine the COFs’ electrochemical behavior in solution, cyclic voltammetry (CV) was carried out with the COFs grown on ITO substrates. The COF‐modified ITO electrodes were exposed to an applied potential ranging from −0.4 to 1.0 V versus ferrocene/ferrocenium (Fc/Fc^+^) inside a three‐electrode cell under argon atmosphere. The cell contained an acetonitrile solution with 1 M tetrabutylammonium hexafluorophosphate (TBA PF_6_) serving as electrolyte. The potential window between −0.4 and 1.0 V versus Fc/Fc^+^ was chosen based on the high stability of the COFs over several cycles within this window at a scan rate of 100 mV s^−1^ (Figure S30, Supporting Information). The CV measurements revealed that both COFs feature reversible redox activity. Distinct (reversible) peaks due to the oxidation of two tertiary amine groups were observed, at a potential of 0.67 and 0.94 V versus Fc/Fc^+^ for the WW COF (Figure 2e) and at 0.62 and 1.00 V versus Fc/Fc^+^ for the WPy‐I COF (Figure 2f) at a scan rate of 100 mV s^−1^. Additionally, the polarization *ΔE* between the oxidative and the reductive waves was remarkably small with 65 mV (WW) and 34 mV (WPy‐I) at scan rates of 50 mV s^−1^. This demonstrates a high reversibility of the redox processes within the ideal reversible range according to the literature.^[^ ^44^ ^]^ At very high scan speeds of 1 V s^−1^, *ΔE* remains small with 180 mV for the WW COF, indicating a high stability and fast redox processes, while the polarization for the WPy‐I COF increases up to 300 mV. This difference suggests enhanced reversibility of the redox centers in the WW structure, owing to the higher density of triphenylamine moieties. Remarkably, the peak shape remains well‐defined even for the WPy‐I COF at 1 V s^−1^. These results indicate that the electrolyte diffusion in the COFs is fast and demonstrates a highly conducting interface between the COF and the current collector.

### 2.3 Stainless Steel Mesh COF Coating

**Figure 3:**
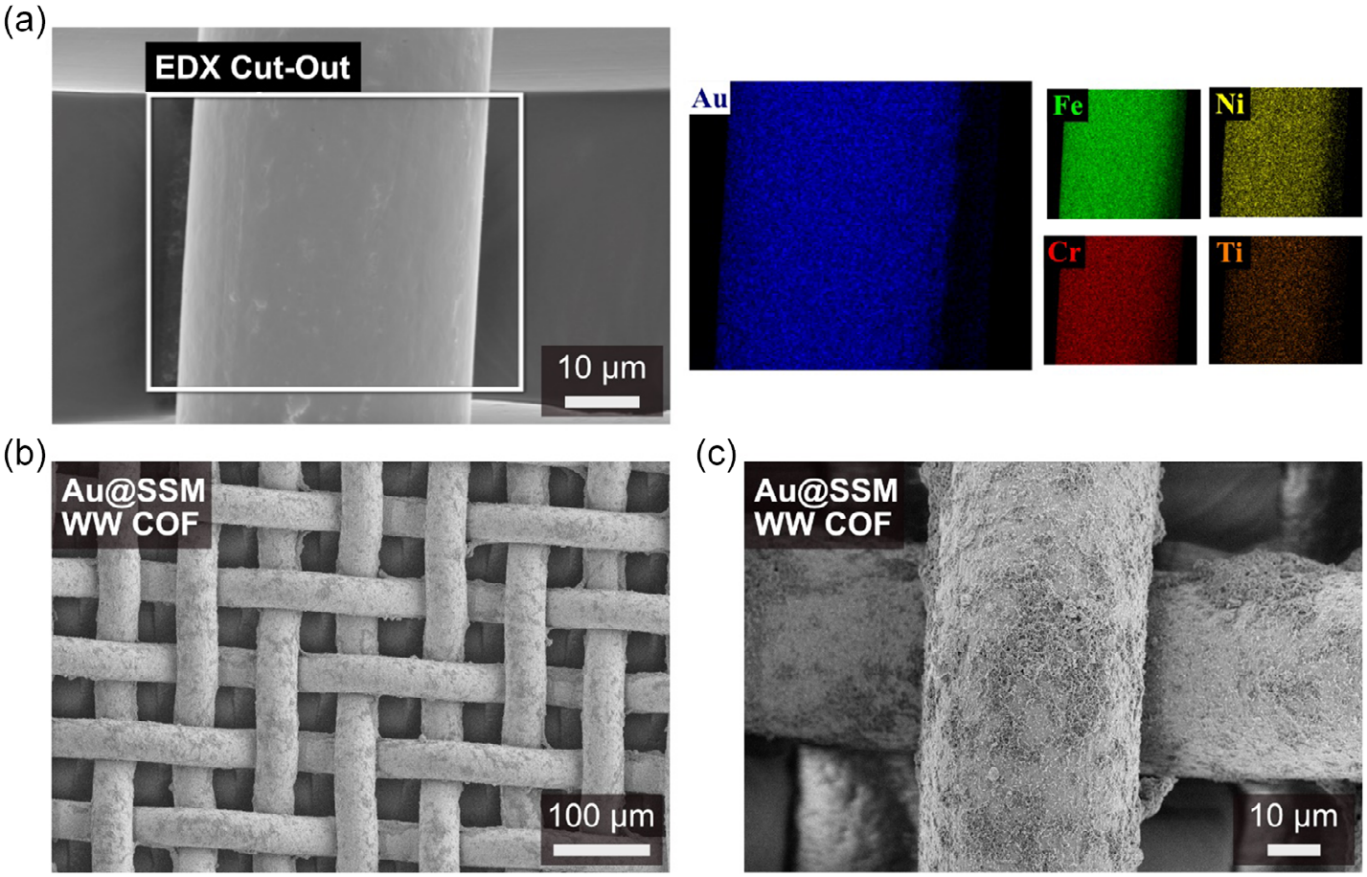
a) SEM image of the Au@SSM with cutout containing color‐coded EDX mapping of the elements gold (dark blue), iron (green), nickel (yellow), chromium (red), and titanium (orange). b,c) SEM images of the WW COF solvothermally grown on an Au@SSM, showing the homogenous distribution of the COF over the whole mesh.

The most commonly used techniques to determine the capacitance of COFs currently involve either using carbon black in a slurry for drop casting or a combination of carbon black and carbon cloth for the device fabrication and the subsequent measurements.^[^ ^8^, ^34^, ^45^, ^46^, ^47^ ^]^ The carbon can thereby significantly enhance the capacitance of the resulting mixture and can compensate the low conductivity of many organic frameworks. However, an important drawback of this approach is that the beneficial impact of carbon itself on the capacitance of the investigated samples frequently remains unaddressed. Here, we implement a novel approach for creating carbon‐free SC devices by coating SSM wires with COFs through solvothermal growth. This process ensures efficient electrical charge transport between the electrode and the electroactive COF by homogeneously binding COF crystallites on the wire surface as a thin layer without any carbon additives. The conductive SSM provides optimal access into the porous network of the COF crystallites and thereby to their high internal surface area and associated significant capacitance. Direct growth of COF crystallites on blank SSMs resulted in a homogenous coverage for the WW COF, but only partially coated the surface with the WPy‐I COF, as evidenced by SEM images (Figure S20 and S21, Supporting Information). A recent study established that a gold coating on SSMs positively impacted the growth of metal–organic frameworks on their surface.^[^ ^48^ ^]^ Inspired by this finding, we pretreated the blank SSMs by depositing a thin layer of 10 nm titanium followed by a 40 nm gold layer using physical vapor deposition (PVD). A wider range of surface growth interfaces was achieved by successfully producing titanium‐, gold‐, silver‐, and aluminum‐coated SSMs. Energy‐dispersive X‐ray (EDX) analysis confirmed the homogeneous coverage of the wires of the mesh (**Figure** **3**a and S19, Supporting Information). This substrate pretreatment method provides a suitable foundation for COF‐based electrodes. For the following COF film synthesis on the different SSMs, the above “standard” film solvothermal synthesis procedure for the WW COF was performed while replacing the film substrate with a 1 cm × 5 cm SSM, which was positioned vertically in the COF reaction vessel (Scheme S1, Supporting Information). Interestingly, for the WPy‐I COF coating, the temperature could be increased to 70 °C while maintaining the frustrated COF formation. PXRD measurements of dislodged powder from the meshes confirmed the successful COF formation (Figure S29, Supporting Information). Only the Ti@SSM did not favor the surface growth of WW COF, which is evident from the reflections of the reactants in the experimental PXRD patterns. Additionally, the Al@SSM significantly decreased the crystallinity of both COFs. SEM examination of the various SSMs was conducted to determine the type and density of the coverage (Figure 3b,c and S22–S28, Supporting Information). The non‐noble metal‐coated SSMs thereby exhibited poor coverage, attributed to possible etching by the acetic acid during the COF reaction. The most homogeneous coverage with the highest crystallinity was achieved on Au@SSM for both COFs, and as such, all subsequent electrochemical measurements were conducted on Au@SSM for comparison.

### 2.4 Device Design and Electrochemical Performance

To calculate the specific capacitance of the COFs without added carbon, the coated SSMs described above offer the advantage of a gravimetric determination of the amount of active material on the mesh, without the need of preparing a COF slurry with additives to afford a suitably conducting electrode. The specific capacitance was then calculated using the areas, scan rates, and potential ranges of obtained CVs together with the mass of the active material (see Section S2, Supporting Information). Primarily, the Au@SSMs with both COFs were tested in acetonitrile, reaching 48.9 F g^−1^ for the WW COF and 7.35 F g^−1^ for the WPy‐I COF (Figure S31, Supporting Information) at a scan rate of 100 mV s^−1^. Due to the doubled density of redox‐active moieties in the WW COF compared to the WPy‐I COF, a higher capacitance was expected for the WW COF. However, the specific ratio between the two capacitances could depend on several additional factors such as inaccessible COF residues between the mesh voids based on the microparticle COF assembly with numerous grain boundaries, different electrical coupling to the gold surface, or the relatively low conductivity of the COFs. To better compare the two COFs with a previously reported isomeric COF, namely the Wurster‐pyrene‐containing TPPDA‐TPPyr COF,^[^ ^34^ ^]^ which achieved a high capacitance value for COFs (see Table S3, Supporting Information), WW and WPy‐I COFs were measured under similar conditions using a carbon slurry (COF:super c65:PVDF = 50:40:10) drop‐cast on a glassy carbon electrode in 0.1 m KOH electrolyte and with a Hg/HgO reference electrode (Figure S32, Supporting Information). Under these conditions, capacitances of 21.2 F g^−1^ (WPy‐I COF) and 30.4 F g^−1^ (WW COF) were obtained. Although WPy‐I did not reach the reported value of 188.7 F g^−1^ (COF:carbon black:Nafion = 45:45:10), the comparison between WW and WPy‐I again confirms the advantageous character of the Wurster motif regarding the capacitance (even with different electrolytes), suggesting that the WW‐COF presents highly promising properties as a SC electrode material.

**Figure 4:**
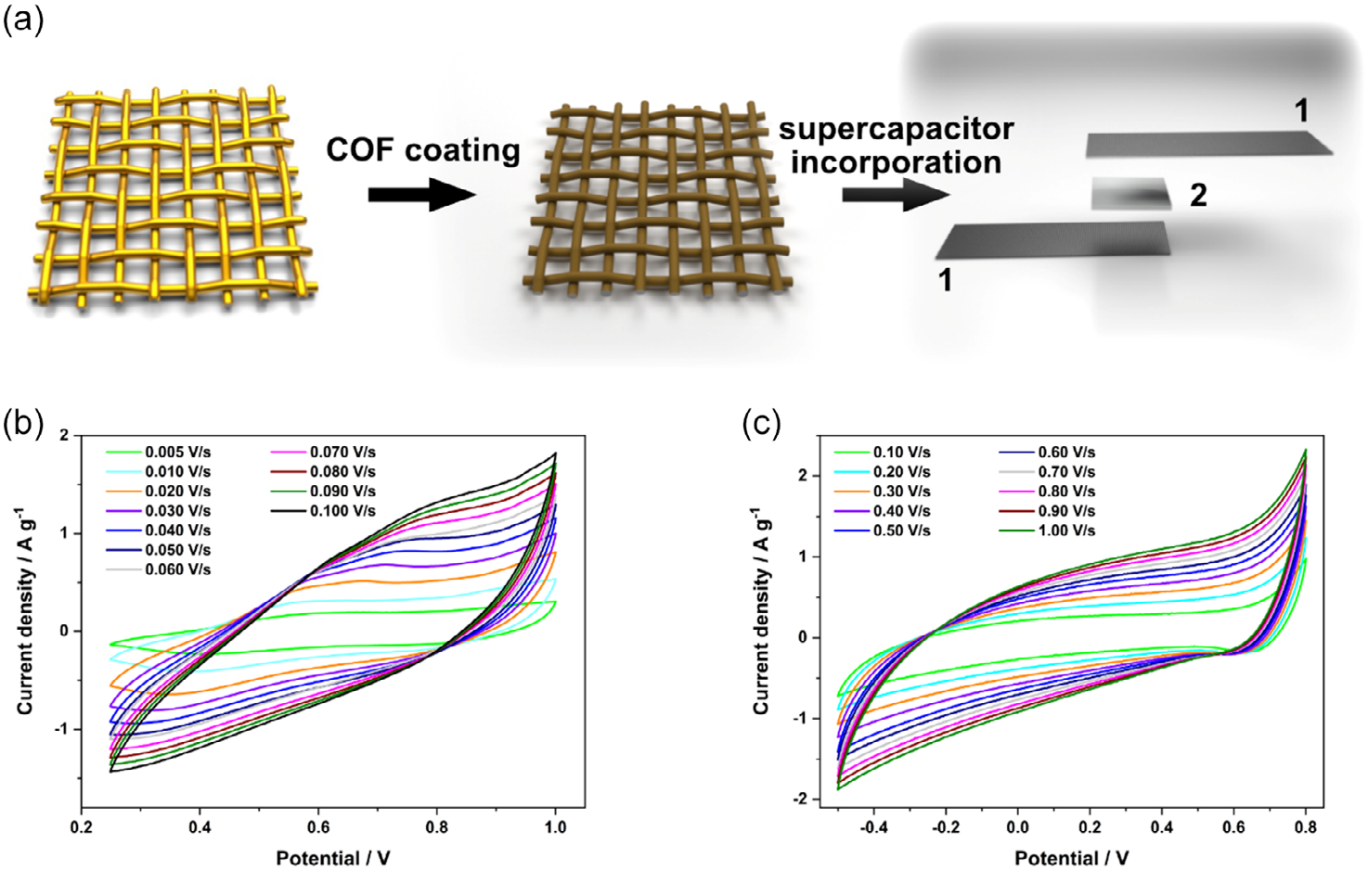
a) A schematic illustration of the Au@SSM coating with COF and the subsequent incorporation of the Au@SSM (1) into a SC device with a separator (2) between the electrodes and the IL 1‐hexyl‐3‐methylimidazolium hexafluorophosphate as electrolyte. CVs of b) WW and c) WPy‐I COF at different scan rates in an Au@SSM device with a glass microfiber separator and the IL electrolyte.

In a next step, both COFs were employed to fabricate symmetric COF devices. In this configuration, the Au@SSMs served as electrodes for a carbon‐free COF SC assembly comprising a glass microfiber mesh as a separator and 1‐hexyl‐3‐methylimidazolium hexafluorophosphate, an ionic liquid (IL), as the electrolyte (**Figure** **4**a). Although ILs have been established as promising candidates for a number of energy‐related applications by virtue of beneficial properties such as low volatility, high ionic conductivity, and high electrochemical and thermal stability,^[^ ^49^, ^50^ ^]^ their usage in combination with COFs has been limited until now.^[^ ^51^, ^52^ ^]^ Having established a COF device configuration with IL electrolyte, we proceeded to examine the electrochemical performance. Thereby, both COFs exhibited CV curve shapes at various scan rates that are close to the rectangular box‐like shape for an ideal SC (Figure 4b,c).^[^ ^53^, ^54^ ^]^ These curve shapes are even maintained at slow scan rates yet with more defined redox peaks, for instance, at a scan rate of 10 mV s^−1^ (Figure S33 and S34, Supporting Information). While for the WPy‐I COF Au@SSM device a capacitance of only 0.90 F g^−1^ was obtained, the WW COF Au@SSM device reached up to 8.85 F g^−1^ at a scan rate of 10 mV s^−1^ (Figure S33 and S34, Supporting Information). To further probe the COF device capacitances and their cycle stability, they were examined by means of galvanostatic charge–discharge (GCD) measurements over 200 cycles at 0.2 A g^−1^ (Figure S35, Supporting Information). The GCD curves display typical triangular shapes with bent regions toward the edges for both devices, implying the existence of electric double‐layer capacitance as well as pseudocapacitance arising from the triphenylamine moieties in the frameworks.^[^ ^55^, ^56^ ^]^ The calculated specific capacitance based on the GCD results revealed a similar trend of the COFs with slightly higher values (WW COF: 11.3 F g^−1^, WPy‐I COF: 4.33 F g^−1^). Considering the cyclability of the devices, a capacitance retention of 80% after the first 20 cycles was observed for both COFs (Figure S36, Supporting Information). This initial loss could be attributed to a detachment of loosely adhered COF particles from the mesh surface. The capacitance remains fairly constant after the initial degradation, with 70.7% (WW COF) and 61.8% (WPy‐I COF) retention of the original capacitance after 200 cycles.

## 3 Conclusion

In summary, here we present COFs based on the electron‐rich Wurster motif, designated as WW COF, WPy‐I COF, and WPy‐II COF. PXRD data demonstrate the high crystallinity of the COFs, serving as the basis for structural simulations of the frameworks, which predict exceptionally small pore sizes. Nitrogen sorption was used to validate the small pore sizes, showcasing the materials’ high porosity. SEM images depict morphologies of intergrown plate‐like particles (WW COF) and spherical agglomerates (WPy‐I COF). These COFs could be homogeneously grown on glass substrates as well as on various SSMs with distinct pre‐coated metal surfaces, thereby yielding a strong (conducting) connection of the active material on the mesh surfaces for electrochemical measurements. Analysis of the redox active COFs via CV revealed that the thin films exhibit reversible redox processes at potential ranges of ≈0.62–0.67 V and 0.94–1.00 V versus Fc/Fc^+^, at a scan rate of 100 mV s^−1^. Small polarization between the oxidative and reductive waves further indicates fast electrolyte diffusion into the COF pores and reversible redox processes. With the homogeneously COF‐covered Au@SSMs, specific capacitance values of up to 7.35 F g^−1^ (WPy‐I COF) and 48.9 F g^−1^ (WW COF) were achieved without additives at 100 mV s^−1^. Furthermore, these coated SSMs emerged as suitable electrodes for symmetrical SC devices that contain only COF particles as active material (no carbon) and an IL as an electrolyte. We emphasize the high capacitance features of these COFs, which are accessible without conductive additives in the presented electrode scenario. Based on the above results, these and related COFs are expected to offer attractive potential toward electrochemical energy storage applications.

## 4 Experimental Section

4.1

4.1.1

##### Synthesis of WW COF

In a 5 mL culture tube, a solid mixture of *N,N,N′,N′*‐tetrakis(4‐aminophenyl)‐1,4‐phenylenediamine (W–NH_2_) (6.5 × 10^−3^ mmol, 3 mg) and *N,N,N′,N′*‐tetrakis(4‐formylphenyl)‐1,4‐phenylenediamine (W–CHO) (6.5 × 10^−3^ mmol, 3.41 mg) was suspended in benzyl alcohol and mesitylene (1 mL, 3:1/v:v). The loading of the culture tube was carried out in an argon‐filled glove box and the resulting dark orange suspension was sonicated in a sonication bath for 1 min at maximum power. Afterward, acetic acid (100 μL, 6 m) was added to the suspension. Subsequently, the culture tube was sealed again, placed in an oven and heated at 100 °C for 4 days. After 4 days, the tube was recovered from the oven and allowed to cool down to room temperature. Subsequently, the resulting dark brown suspension was collected, washed with dry THF (5 mL), and dried at 120 °C under dynamic vacuum. Finally, a chemical extraction with supercritical carbon dioxide (100 bar, 40 °C) as solvent yielded the WW COF as a reddish brown powder (4.30 mg, 72%).

##### Synthesis of WPy‐I COF

In a 5 mL culture tube, a solid mixture of 1,3,6,8‐tetrakis(4‐aminophenyl)pyrene (Py–NH_2_) (6.5 × 10^−3^ mmol, 4.26 mg) and W–CHO (6.5 × 10^−3^ mmol, 3.41 mg) was suspended in benzyl alcohol and mesitylene (1 mL, 1:1/v:v). Afterward, aniline (9 equiv.) was added, serving as synthesis modulator. The loading of the culture tube was carried out in an argon‐filled glove box and the resulting orange suspension was sonicated in a sonication bath for 1 min at maximum power. Subsequently, acetic acid (100 μL, 6 m) was added to the suspension and the culture tube was placed int a preheated oven at 50 °C for 5 days. After 5 days, the tube was recovered from the oven and allowed to cool down to room temperature. Subsequently, the resulting slightly orange suspension was collected, washed with dry THF (5 mL) and dried at 120 °C under dynamic vacuum. The crude product was obtained as a light orange powder (6.24 mg, 88%).

##### Synthesis of WW COF thin Films

In a 50 mL laboratory bottle, a solid mixture of W–CHO (19.5 × 10^−3^ mmol, 10.23 mg) and W–NH_2_ (19.5 × 10^−3^ mmol, 9 mg) was dissolved in benzyl alcohol and mesitylene (3 mL, 3:1/v:v). A substrate holder with a horizontally orientated glass or ITO substrate (face down) was placed into the laboratory bottle and acetic acid (300 μL, 6 m) was added. The solvothermal reaction was performed at 100 °C for 5 days in an oven. The reaction ended by removing the reactor from the oven, followed by cooling to room temperature. The substrates were recovered and were worked up by washing with THF (10 mL), cleaning the upper side of the substrates with ethanol, and the obtained brownish films were dried with compressed air.

##### Synthesis of WPy‐I COF Thin Films

In a 50 mL laboratory bottle, a solid mixture of W–CHO (19.5 × 10^−3^ mmol, 10.23 mg) and Py–NH_2_ (19.5 × 10^−3^ mmol, 12.78 mg) was dissolved in benzyl alcohol and mesitylene (3 mL, 1:1/v:v). A substrate holder with a horizontally orientated glass or ITO substrate (face down) was placed into the laboratory bottle and acetic acid (300 μL, 6 m) was added. The solvothermal reaction was performed at 50 °C for 5 days in an oven. The reaction ended by removing the reactor from the oven, followed by cooling to room temperature. The substrates were recovered and were worked up by washing with THF (10 mL), cleaning the upper side of the substrates with ethanol, and the obtained yellow films were dried with compressed air.

##### Synthesis of WW COF‐Coated (Ti/Au/Ag/Al)@)SSMs

In a 50 mL laboratory bottle, a solid mixture of W–CHO (19.5 × 10^−3^ mmol, 10.23 mg) and W–NH_2_ (19.5 × 10^−3^ mmol, 9 mg) was dissolved in benzyl alcohol and mesitylene (3 mL, 3:1/v:v). A substrate holder with a vertically orientated 1 cm × 5 cm (Ti/Au/Ag/Al)@)SSM was placed into the laboratory bottle such that an area of 1 cm^2^ of the SSM was immersed into the reaction solution (see Scheme S1, Supporting Information). The remaining substrate was used for stabilization and later for contacting the active area. Subsequently, acetic acid (300 μL, 6 m) was added. The solvothermal reaction was performed at 90 °C for 5 days in an oven. The SSM was recovered and was worked up by washing with THF (20 mL) to remove the reactants and all COF residues between the mesh wires. The contact area was shortened and cleaned with ethanol, and the obtained WW‐coated SSM was dried under dynamic vacuum. As a result, a COF loading of 0.5–2 mg on the SSM substrates (width × length = 1 cm × 1 cm) was achieved, depending on the metal coating.

##### Synthesis of WPy‐I COF‐Coated (Ti/Au/Ag/Al)@)SSMs

In a 50 mL laboratory bottle, a solid mixture of W–CHO (19.5 × 10^−3^ mmol, 10.23 mg) and Py–NH_2_ (19.5 × 10^−3^ mmol, 12.78 mg) was dissolved in benzyl alcohol and mesitylene (3 mL, 1:1/v:v). A substrate holder with a vertically orientated 1 cm × 5 cm (Ti/Au/Ag/Al)@)SSM was placed into the laboratory bottle so that an area of 1 cm^2^ of the SSM was immersed into the reaction solution (see Scheme S1, Supporting Information). The remaining substrate was used for stabilization and later for contacting the active area. Subsequently, acetic acid (300 μL, 6 m) was added. The solvothermal reaction was performed at 70 °C for 5 days in an oven. The SSM was recovered and was worked up by washing with THF (20 mL) to remove the reactants and all COF residues between the mesh wires. The contact area was shortened and cleaned with ethanol, and the obtained WPy‐coated SSM was dried under dynamic vacuum. As a result, a COF loading of 0.5–2 mg on the SSM substrates (width × length = 1 cm × 1 cm) was achieved, depending on the metal coatin.

## Conflict of Interest

The authors declare no conflict of interest.

## Author Contributions

**Roman Guntermann**: conceptualization (equal); data curation (lead); formal analysis (lead); investigation (lead); methodology (lead); project administration (supporting); validation (lead); visualization (lead); writing—original draft (lead); writing—review and editing (equal). **Julian M. Rotter**: conceptualization (equal); data curation (supporting); formal analysis (supporting); investigation (supporting); methodology (supporting); project administration (supporting); supervision (supporting); validation (supporting). **Apeksha Singh**: data curation (supporting); formal analysis (supporting); investigation (supporting); validation (supporting); visualization (supporting); writing—original draft (supporting); writing—review and editing (supporting). **Dana D. Medina**: conceptualization (supporting); formal analysis (supporting); project administration (supporting); supervision (supporting); validation (supporting); visualization (supporting); writing—original draft (supporting); writing—review and editing (supporting). **Thomas Bein**: conceptualization (equal); funding acquisition (lead); project administration (lead); resources (lead); software (lead); supervision (lead); validation (lead); writing—review and editing (equal).

## Supporting information

Supplementary Material

## Data Availability

The data that support the findings of this study are available from the corresponding author upon reasonable request.
