# Supplementary material for: Redox‐Active Microporous Covalent Organic Frameworks for Additive‐Free Supercapacitors
Source: Small Sci. 2025 Mar 21;5(6):2400585. doi: 10.1002/smsc.202400585 (PMC12168591; doi:10.1002/smsc.202400585)
Supplement: Supplementary file 1 — Supplementary Material [file SMSC-5-2400585-s001.pdf]

Supporting Information

Redox-active Microporous Covalent Organic Frameworks for Additive-free Supercapacitors

*Roman Guntermann,[1] Julian M. Rotter,[1] Apeksha Singh,[1] Dana D. Medina,[1] Thomas Bein[1,]**

[1]Department of Chemistry and Center for NanoScience (CeNS), University of Munich (LMU), Butenandtstraße 5-13, 81377 Munich, Germany

S1. Methods 2

S2. Electrochemical characterization 4

S3. Building block synthesis 5

S4. COF bulk synthesis 7

S5. COF thin film synthesis 10

S6. COF coated SSM synthesis 11

S7. FTIR spectroscopy 12

S8. Structural analysis 12

S9. Nitrogen sorption 17

S10. Thermogravimetric Analysis 17

S11. Scanning Electron Microscopy of COF bulk and films 18

S12. Transmission Electron Microscopy 19

S13. Conductivity 19

S14. Grazing incidence wide angle X-ray scattering 20

S15. Optical properties 20

S16. EDX mapping of the Ti/Ag/Al@SSMs 21

S17. Scanning Electron Microscopy of COF coated SSMs 22

S18. XRD of the SSM active material 25

S19. Electrochemical characterization 25

S20. Electrochemical reference measurements 33

S21. References 57

S1. Methods

General: All materials were purchased from Aldrich, Acros or TCI Europe in the common purities purum, puriss or reagent grade. Materials were used as received without additional purification and handled in air unless denoted. All solvents used were anhydrous and purged with inert gas. Whatman® Glass microfiber filters used as separators were purchased from Aldrich with a diameter of 55 mm and cut to the required shape. SSMs were purchased from Metallwaren-Riffert with a wire thickness of 0.035 mm and mesh size of 0.05 mm.

Nuclear magnetic resonance (NMR) spectra were recorded on Bruker AV 400 and AV 400 TR spectrometers. Chemical shifts are expressed in parts per million (δ scale) and are calibrated using residual (undeuterated) solvent peaks as an internal reference (^{1}H-NMR: $CDCl_3$: 7.26, DMSO-d_6: 2.50, DMF-d_7: 8.03; ^{13}C-NMR: $CDCl_3$: 77.16, DMSO-d_6: 39.52, DMF-d_7: 163.15). Data for ^{1}H NMR spectra are reported in the following way: chemical shift (δ, ppm) (multiplicity, coupling constant/ Hz, integration). Multiplicities are reported as follows: s = singlet, d = doublet, t = triplet, q = quartet, m = multiplet, or combinations of thereof.

High resolution electron ionization (EI) **mass spectra** (MS) were recorded with a Thermo Finnigan MAT 95 instrument.

Fourier-transform infrared spectroscopy (FTIR) measurements were performed with a Bruker Vertex 70 FTIR instrument by focusing light of a globar (silicon carbide) as MIR light source through a KBr beam splitter with integrated gold mirrors and an ATR sample stage with a Ge crystal. The spectra were recorded with a N_2 cooled MCT detector at a resolution of 2 cm^{-1} and averaged over 500 scans.

Powder X-ray diffraction measurements were performed on a Bruker D8 Discover diffractometer using Ni-filter Cu K_α radiation and a position sensitive LynxEye detector in Bragg-Brentano geometry.

The **structure models of the COFs** were constructed using the Accelrys Materials Studio software package. For each COF the highest possible symmetry/space group was applied. The structure models were optimized using the Forcite module with the Universal force-field. Structure refinements using the Pawley method were carried out as implemented in the Reflex module of the Materials Studio software. Thompson-Cox-Hastings peak profiles were used, and peak asymmetry was corrected using the Berar-Baldinozzi method.

2D grazing-incidence wide angle X-ray scattering (GIWAXS) data were recorded with an Anton Paar SAXSpoint 2.0 system equipped with a Primux 100 micro Cu K_α source and a Dectris EIGER R 1M 2D detector. The COF films were positioned at a sample-detector distance of 140 mm and were measured with an incidence angle of 0.2°

Nitrogen sorption isotherms were recorded on a Quantachrome Autosorb 1 instrument at 77 K within pressure ranges of p/p_0 = 0.001 to 0.98. Prior to the measurements, the samples were heated for 12 h at 100 °C under high vacuum. For the evaluation of the surface area, the BET method was applied within a p/p_0 range of 0.05 to 0.3. Pore size distributions were calculated using the QSDFT absorption model with a carbon kernel for cylindrical pores.

Thermogravimetric analysis (TGA) measurements were performed on a Netzsch Jupiter ST 499 C instrument equipped with a Netzsch TASC 414/4 controller. The samples were heated from room temperature to 900 °C under a synthetic air flow (25 ml min^{-1}) at a heating rate of 10 K min^{-1}.

UV-VIS-NIR spectra were recorded using a Perkin-Elmer Lambda 1050 spectrometer equipped with a 150 mm integrating sphere, photomultiplier tube (PMT) and InGaAs detector.

Scanning electron microscopy images were recorded with an FEI Helios NanoLab G3 UC scanning electron microscope equipped with a field emission gun operated at 3 kV. Prior to the measurements, the samples were sputtered with carbon.

Transmission electron microscopy images were recorded with an FEI Titan Themis 60 – 300 instrument equipped with a field emission gun operated at 300 kV. The samples were prepared by placing them onto a copper grid supporting a thin, electron transparent carbon film.

Van der Pauw measurements were carried out at room temperature (292 K) using an ECOPIA Model HMS-5300 Hall measurement setup. Gold contact electrodes were placed in a square geometry with distances of 2.4 mm on pressed pellets of the crystalline samples. Powder pellets were pressed by using approximately 15 mg of COF material and pressing it into a cylindrical pellet with a diameter of 1 cm under a pressure of 8 MPa. Pellet thicknesses were measured with a slide gauge to be about 200 μm.

(Ti/Au/Ag/Al@)SSMs were prepared by cutting SSMs into 18 x 6 cm large pieces and cleaning by ultrasonic treatment in acetone followed by ethanol and water. SSMs were positioned in a rotating vacuum deposition unit installed in a glovebox (MBraun Labmaster Pro SP equipped with an Inficon SQC-310C deposition controller). For the Ti@SSMs, a 10 nm thin film of titanium was thermally deposited under high vacuum on the mesh. For the Au@SSMs, a 10 nm thin film of titanium as adhesion layer and subsequently a 40 nm gold thin film were thermally deposited under high vacuum on the mesh. For the Ag@SSMs, a 10 nm thin film of titanium as adhesion layer and subsequently a 40 nm silver thin film were thermally deposited under high vacuum on the mesh. For the Al@SSMs, a 60 nm thin film of aluminum was thermally deposited under high vacuum on the mesh. The evaporation procedure was conducted for both sides of each SSM. For the COF coating of the meshes, the (Ti/Au/Ag/Al@)SSMs were cut into 1 cm x 5 cm pieces and cleaned under nitrogen flow prior to solvothermal synthesis.

S2. Electrochemical characterization

Electrochemical measurements were performed on a Metrohm µAutolabIII/FRA2 instrument using a three-electrode setup with a Pt-wire counter-electrode and a silver wire as a pseudo reference electrode under argon atmosphere. Film and SSMs measurements were carried out in dry acetonitrile with tetrabutyl ammonium tetrafluoroborate (1 M) serving as the electrolyte. After recording CV data, the measurement was repeated with the addition of a small amount of ferrocene to the electrolyte solution. The position of the Fc/Fc^+ redox couple potential was then used as the reference potential. Cyclic voltammetry measurements on a glassy carbon electrode (GCE) were performed by drop-casting 30 µL of a *N*-methylpyrrolidone (NMP) slurry containing 50% COF, 40% super c65 carbon black obtained from Targray and 10% polyvinylidene fluoride (PVDF) binder on the GCE. The electrodes were dried at 60 °C for 72 h and afterwards measured in 0.1 M potassium hydroxide electrolyte. The SSM devices were measured with the COF serving as active material on two SSMs with a glass microfiber in between as separator and the ionic liquid 1-hexyl-3-methylimidazolium hexafluorophosphate as electrolyte. Using this setup, cyclic voltammetry as well as galvanostatic charge-discharge (GCD) cycle tests were performed. Reference measurements were performed using an Au@SSM device without COF coating in 1-hexyl-3-methylimidazolium hexafluorophosphate electrolyte, revealing negligible currents (see Figure S37).

Electrochemical Calculations

The specific capacitance C_P was calculated based on Equation S1 for the cyclic voltammetry measurements and based on Equation S2 for galvanostatic charge-discharge measurements:[1,2]

$$C_P = \frac{A}{2mv(V_2 - V_1)} \qquad \text{(S1)}$$

A = area inside the CV curve (A·V)
m = mass of the electroactive material (g)
v = scan rate (V/s)
$V_2 - V_1$ = potential window (V)

$$C_P = \frac{I\Delta t}{\Delta V m} \qquad \text{(S2)}$$

I = current (A)
Δt = discharging time (s)
ΔV = potential window (V)
m = mass of the electroactive material (g)

S3. Building block synthesis

Synthesis of *N,N,N',N'*-tetrakis(4-formylphenyl)-1,4-phenylenediamine (W–CHO)

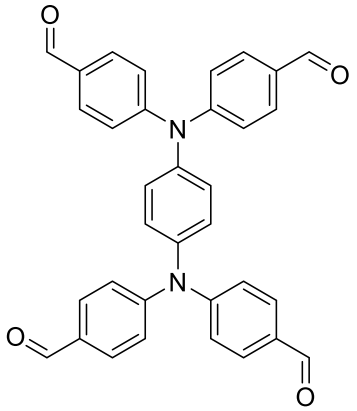

N,N,N',N'-tetraphenyl-1,4-phenylenediamine (1.55 g, 3.76 mmol, 1.0 equiv.) and imidazole (1.98 g, 29.1 mmol, 7.7 equiv.) were dissolved in CH_3CN (150 mL), and trifluoroacetic anhydride (12.2 g, 58.2 mmol, 15.4 equiv.) was added. The resulting reaction mixture was heated to reflux for 16 h and, after cooling to room temperature, poured into ice water (200 mL). The precipitated green solid was isolated by filtration, washed with water and dissolved in CH_3CN (150 mL). 2 M HCl (50 mL) was added and the reaction was heated to 100 °C for 16 h. After cooling to room temperature, the mixture was poured into ice water (200 mL). The precipitated brown product was filtrated off and washed with water. The crude product was purified by stirring in a boiling mixture of cyclohexane and chloroform followed by filtration and a purification via column chromatography (silica gel, DCM/Ethyl acetate 5:1) to give *N,N,N',N'*-tetrakis(4-formylphenyl)-1,4-phenylenediamine (1.14 g, 2.17 mmol, 58%) as a bright yellow solid.

^{1}H NMR (400 MHz, $CDCl_3$): δ (ppm) = 9.92 (s, 4H), 7.82 (d, J = 8.8 Hz, 8H), 7.24 (d, J = 8.8 Hz, 8H), 7.17 (s, 4H).

^{13}C NMR (100 MHz, $CDCl_3$): δ (ppm) = 190.2, 151.5, 142.8, 131.5, 131.3, 127.7, 123.0.

HRMS (EI-orbitrap): *m/z*: [M] calc. for [$C_{34}H_{24}N_2O_4$]: 524.17; found 524.47.

Elemental analysis (calculated, found for $C_{34}H_{24}N_2O_4$): C (77.85, 75.92), H (4.61, 4.97), N (5.34, 4.92).

Synthesis of 1,3,6,8-tetrakis(4-aminophenyl)pyrene (Py–NH_2)

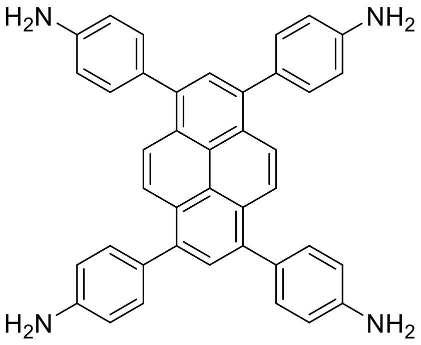

Based on a reported recipe,[3] 1,3,6,8-tetrabromopyrene (494 mg, 0.95 mmol, 1.0 eq.), 4-aminophenylboronic acid pinacol ester (1000 mg, 4.6 mmol, 4.8 eq.), K_2CO_3 (725 mg, 5.2 mmol, 5.5 eq.) and $Pd(PPh_3)_4$ (110 mg, 0.10 mmol, 10 mol%) were mixed in 11 mL 1,4-dioxane and 2.7 mL H_2O. The mixture was heated to reflux (115 °C) for 3 d. After cooling to room temperature, H_2O (30 mL) was added. The resulting precipitate was collected via filtration, washed with H_2O and MeOH and recrystallized from 1,4-dioxane. After drying under high vacuum, the title compound was obtained, co-crystallized with approximately 1.5 dioxane molecules per formula unit, as a brown-yellow powder (559 mg, 0.80 mmol, 84%).

^{1}H NMR (400 MHz, DMSO-*d_6*): δ (ppm) = 8.13 (s, 4H), 7.79 (s, 2H), 7.34 (d, J = 8.4 Hz, 8H), 6.77 (d, J = 8.5 Hz, 8H), 5.30 (s, 8H), 3.56 (s, 12 H, dioxane).

S4. COF bulk synthesis

Synthesis of WW COF

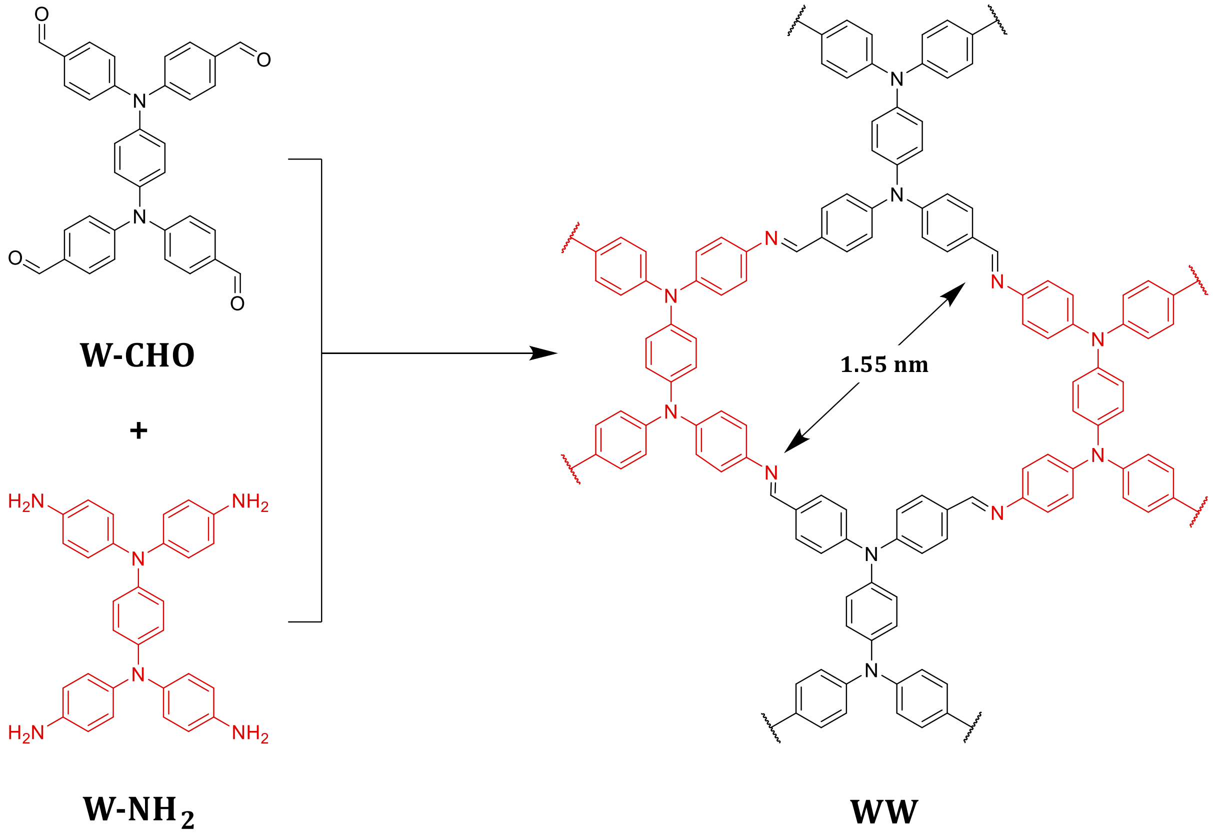

In a 5 mL culture tube, a solid mixture of *N,N,N',N'*-tetrakis(4-aminophenyl)-1,4-phenylenediamine (W–NH_2) (6.5×10^{-3} mmol, 3 mg) and *N,N,N',N'*-tetrakis(4-formylphenyl)-1,4-phenylenediamine (W–CHO) (6.5×10^{-3} mmol, 3.41 mg) was suspended in 1 mL benzyl alcohol and mesitylene (3:1 / v:v). The loading of the culture tube was carried out in an argon filled glove box and the resulting dark orange suspension was sonicated in a sonication bath for 1 min at maximum power. Afterwards, 100 µL of acetic acid (6 M) were added to the suspension. Subsequently, the culture tube was sealed again and placed in an oven and preheated at 100 °C for 4 days. After 4 days, the tube was recovered from the oven and allowed to cool down to room temperature. Subsequently, the resulting dark brown suspension was collected, washed with 5 mL of dry tetrahydrofurane (THF) and dried at 120 °C under dynamic vacuum. Finally, a chemical extraction with supercritical carbon dioxide (100 bar, 40 °C) as solvent yielded a reddish product. The WW COF was obtained as a reddish brown powder (4.30 mg, 72%).

Elemental analysis (calculated, found for $C_{64}H_{44}N_8$): C (83.09, 78.54), H (4.79, 4.49), N (12.11, 10.93).

Synthesis of WPy-I COF

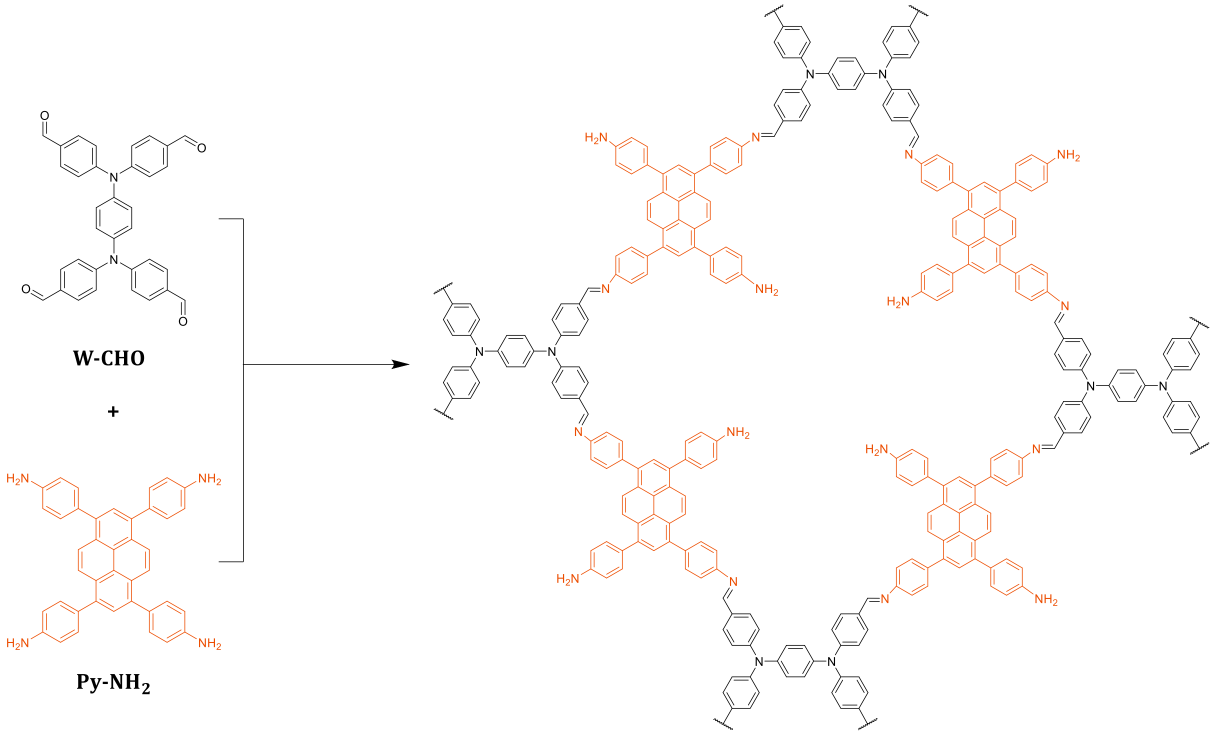

WPy-I

In a 5 mL culture tube, a solid mixture of 1,3,6,8-tetrakis(4-aminophenyl)pyrene (Py–NH_2) (6.5×10^{-3} mmol, 4.26 mg) and W–CHO (6.5×10^{-3} mmol, 3.41 mg) was suspended in 1 mL benzyl alcohol and mesitylene (1:1 / v:v). Afterwards, aniline (9 equiv.) was added serving as synthesis modulator.[4] The loading of the culture tube was carried out in an argon filled glove box and the resulting orange suspension was sonicated in a sonication bath for 1 min at maximum power. Subsequently, 100 µL acetic acid (6 M) were added to the suspension and the culture tube was placed in a preheated oven at 50 °C for 5 days. After 5 days, the tube was recovered from the oven and allowed to cool down to room temperature. Subsequently, the resulting slightly orange suspension was collected, washed with 5 mL of dry THF and dried at 120 °C under dynamic vacuum. The crude product was obtained as a light orange powder (6.24 mg, 88%).

Elemental analysis (calculated, found for $C_{114}H_{76}N_{10}$): C (86.34, 82.21), H (4.83, 4.89), N (8.83, 7.14).

Synthesis towards WPy-II COF

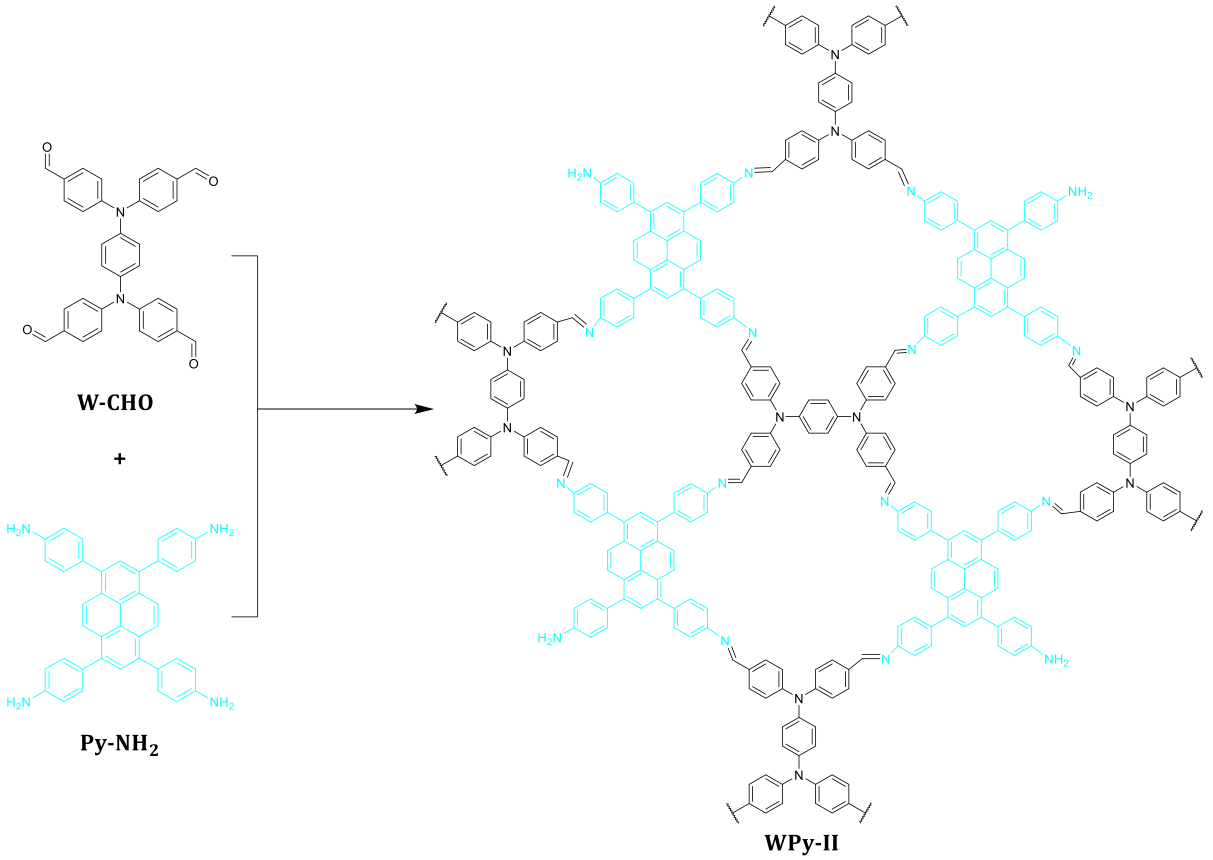

Since a mere pattern of a fully bonded framework could not be achieved, we state following synthesis protocol as method 'towards' WPy-II COF with WPy-I residuals. In a 5 mL culture tube, a solid mixture of 1,3,6,8-tetrakis(4-aminophenyl)pyrene (Py–NH_2) (6.5×10^{-3} mmol, 4.26 mg) and W–CHO (6.5×10^{-3} mmol, 3.41 mg) was suspended in 1 mL benzyl alcohol and mesitylene (1:1 / v:v). Afterwards, aniline (9 equiv.) was added serving as synthesis modulator.[4] The loading of the culture tube was carried out in an argon filled glove box and the resulting orange suspension was sonicated in a sonication bath for 1 min at maximum power. Subsequently, 100 µL acetic acid (6 M) were added to the suspension and the culture tube was placed in a preheated oven at 120 °C for 5 days. After 5 days, the tube was recovered from the oven and allowed to cool down to room temperature. Subsequently, the resulting slightly orange suspension was collected, washed with 5 mL of dry THF and dried at 120 °C under dynamic vacuum. The crude product was obtained as a yellow powder (4.47 mg, 63%).

Elemental analysis (calculated, found for $C_{74}H_{46}N_6$): C (87.20, 80.77), H (4.55, 5.01), N (8.25, 6.14).

S5. COF thin film synthesis

Synthesis of WW COF thin films

In a 50 mL laboratory bottle, a solid mixture of W–CHO (19.5 × 10^{-3} mmol, 10.23 mg) and W–NH_2 (19.5 × 10^{-3} mmol, 9 mg) was dissolved in 3 mL benzyl alcohol and mesitylene (3:1 / v:v). A substrate holder with a horizontally orientated glass or indium tin oxide (ITO) substrate was placed into the laboratory bottle and 300 µL of acetic acid (6 M) were added. The solvothermal reaction was performed at 100 °C for 5 days in an oven. The reaction ended by removing the reactor from the oven, followed by cooling to room temperature. The substrates were recovered and were worked up by washing with 10 mL THF, cleaning the upper side of the substrates with ethanol, and the obtained brownish films were dried with compressed air.

Synthesis of WPy-I COF thin films

In a 50 mL laboratory bottle, a solid mixture of W–CHO (19.5 × 10^{-3} mmol, 10.23 mg) and Py–NH_2 (19.5 × 10^{-3} mmol, 12.78 mg) was dissolved in 3 mL benzyl alcohol and mesitylene (1:1 / v:v). A substrate holder with a horizontally orientated glass or ITO substrate was placed into the laboratory bottle and 300 µL of acetic acid (6 M) were added. The solvothermal reaction was performed at 50 °C for 5 days in an oven. The reaction ended by removing the reactor from the oven, followed by cooling to room temperature. The substrates were recovered and were worked up by washing with 10 mL THF, cleaning the upper side of the substrates with ethanol, and the obtained yellow films were dried with compressed air.

S6. COF coated SSM synthesis

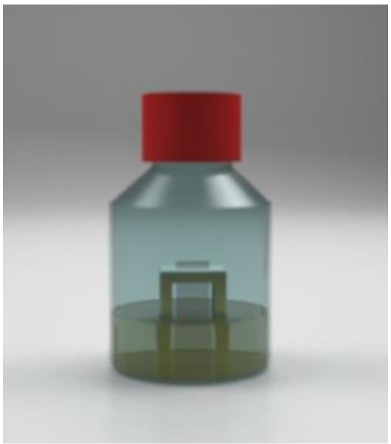

Scheme S1. A schematic illustration of the SSM COF synthesis.

Synthesis of WW COF coated (Ti/Au/Ag/Al)@)SSMs

In a 50 mL laboratory bottle, a solid mixture of W–CHO (19.5 × 10^{-3} mmol, 10.23 mg) and W–NH_2 (19.5 × 10^{-3} mmol, 9 mg) was dissolved in 3 mL benzyl alcohol and mesitylene (3:1 / v:v). A substrate holder with a vertically orientated 1 cm x 5 cm (Ti/Au/Ag/Al)@)SSM was placed into the laboratory bottle so that an area of 1 cm^2 of the SSM was immersed into the reaction solution (see Scheme S1). The remaining substrate was used for stabilisation and later for contacting the active area. Subsequently, 300 µL of acetic acid (6 M) were added. The solvothermal reaction was performed at 90 °C for 5 days in an oven. The SSM was recovered and was worked up by washing with 20 mL THF to remove the reactants and all COF residues between the mesh wires. The contact area was shortened and cleaned with ethanol, and the obtained WW coated SSM was dried under dynamic vacuum. As a result, a COF loading of 0.5-2 mg on the SSM substrates (width × length = 1 cm × 1 cm) was achieved.

Synthesis of WPy-I COF coated (Ti/Au/Ag/Al)@)SSMs

In a 50 mL laboratory bottle, a solid mixture of W–CHO (19.5 × 10^{-3} mmol, 10.23 mg) and Py–NH_2 (19.5 × 10^{-3} mmol, 12.78 mg) was dissolved in 3 mL benzyl alcohol and mesitylene (1:1 / v:v). A substrate holder with a vertically orientated 1 cm x 5 cm (Ti/Au/Ag/Al)@)SSM was placed into the laboratory bottle so that an area of 1 cm^2 of the SSM was immersed into the reaction solution (see scheme S1). The remaining substrate was used for stabilisation and later for contacting the active area. Subsequently, 300 µL of acetic acid (6 M) were added. The solvothermal reaction was performed at 70 °C for 5 days in an oven. The SSM was recovered and was worked up by washing with 20 mL THF to remove the reactants and all COF residues between the mesh wires. The contact area was shortened and cleaned with ethanol, and the obtained WPy coated SSM was dried under dynamic vacuum. As a result, a COF loading of 0.5-2 mg on the SSM substrates (width × length = 1 cm × 1 cm) was achieved.

S7. FTIR spectroscopy

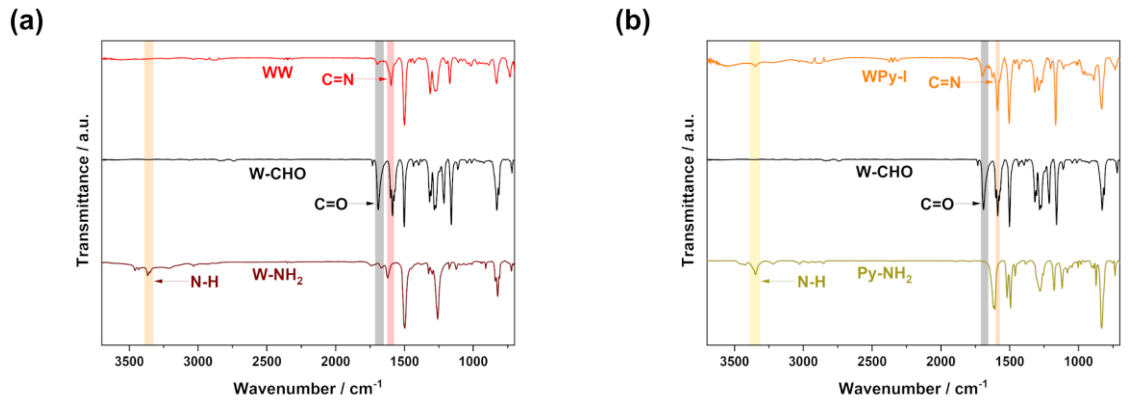

Figure S1. Comparison of the FTIR spectra of the amine building blocks W−NH_2 (dark red) and Py−NH_2 (yellow), the aldehyde precursor W−CHO (black) and the resulting (**a**) WW and (**b**) WPy-I COFs in red and orange, respectively. The significant bands for each compound are marked and assigned to the corresponding functional groups.

S8. Structural analysis

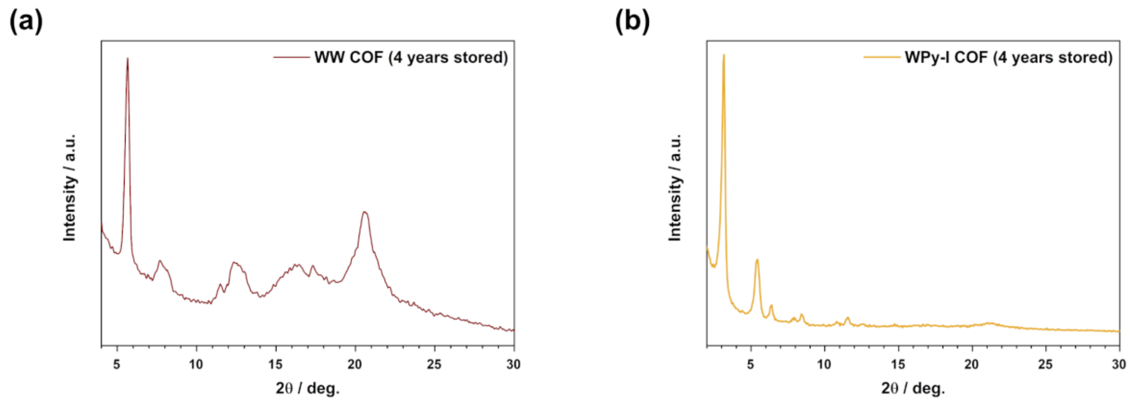

Figure S2. XRD pattern of the (**a**) WW and (**b**) WPy-I COFs remeasured after four years while stored under atmospheric conditions.

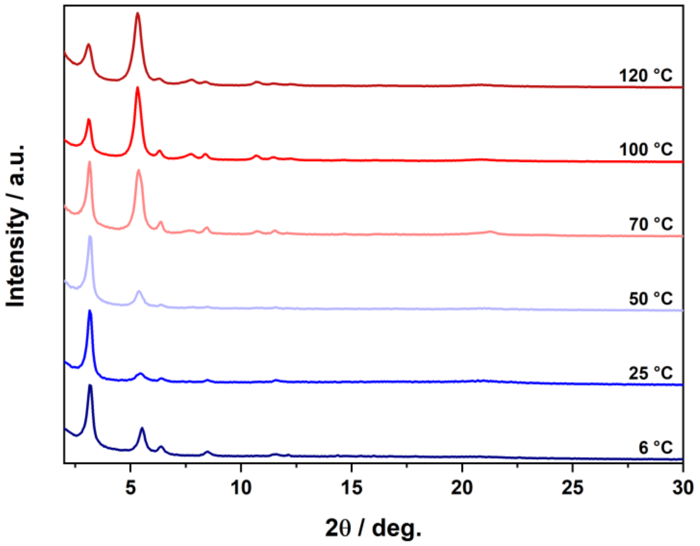

Figure S3. Powder XRD patterns of the WPy based COFs synthesized at different temperatures.

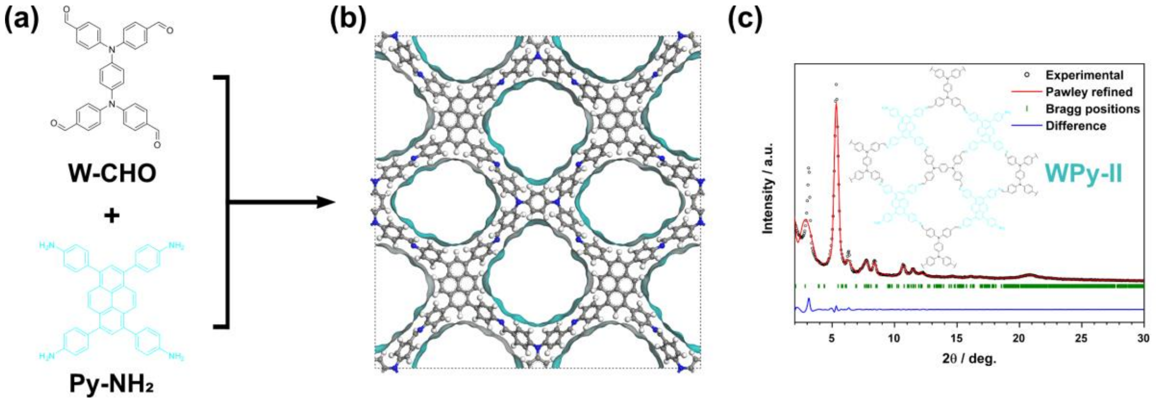

Figure S4. (**a**) Schematic presentation of the synthesis of WPy-II COF by an imine condensation with a Pawley refined structure of the COF (**b**). (**c**) Experimental PXRD pattern (black dots) of WPy-II COFs. The Pawley refinement (red line), the difference plot between the experimental data and the Pawley-refined PXRD pattern (blue line) are shown, and the Bragg positions are indicated by green ticks. A schematic representation of the WPy-II COF is shown as inset.

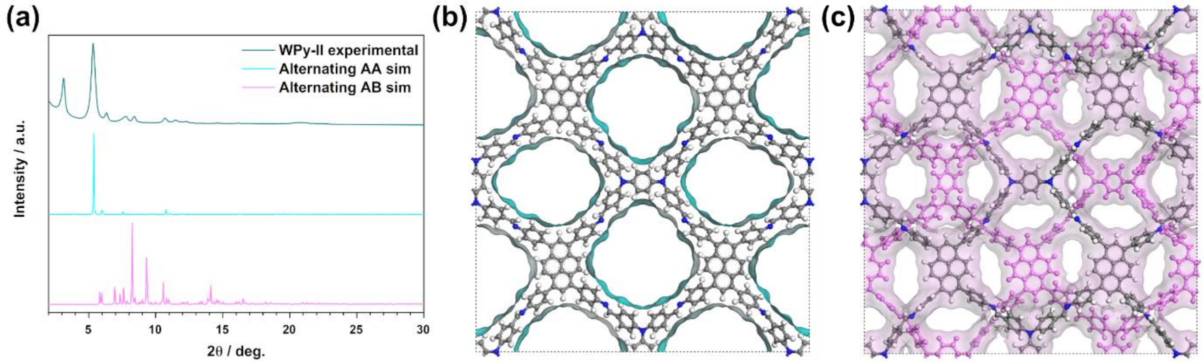

Figure S5. (**a**) Experimental powder XRD pattern of a synthesis 'towards' WPy-II COF (turquoise) and simulated PXRD patterns for eclipsed (blue) and staggered structure (pink) of an alternating building block arrangement. Respective simulated crystal structures of (**b**) eclipsed structure and (**c**) staggered structure.

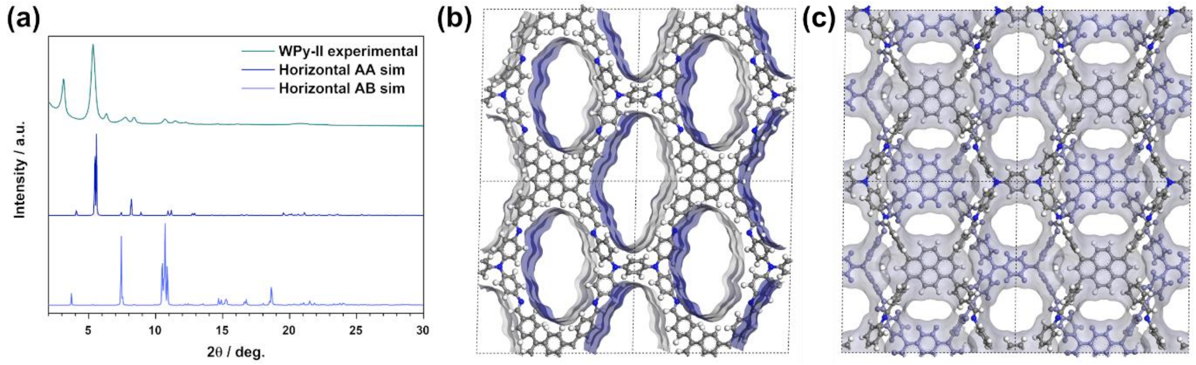

Figure S6. (**a**) Experimental powder XRD pattern of a synthesis 'towards' WPy-II COF (turquoise) and simulated PXRD patterns for eclipsed (navy) and staggered structure (light blue) of a horizontal building block arrangement. Respective simulated crystal structures of (**b**) eclipsed structure and (**c**) staggered structure.

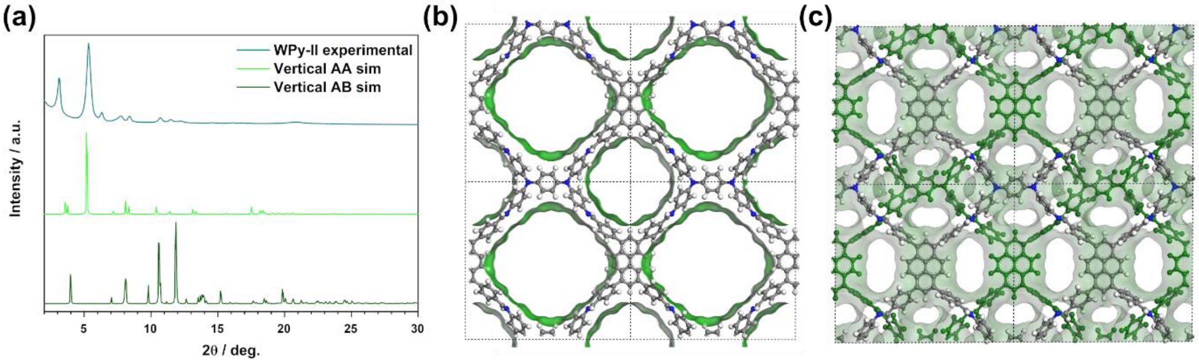

Figure S7. (**a**) Experimental powder XRD pattern of a synthesis 'towards' WPy-II COF (turquoise) and simulated PXRD patterns for eclipsed (light green) and staggered structure (dark green) of a vertical building block arrangement. Respective simulated crystal structures of (**b**) eclipsed structure and (**c**) staggered structure.

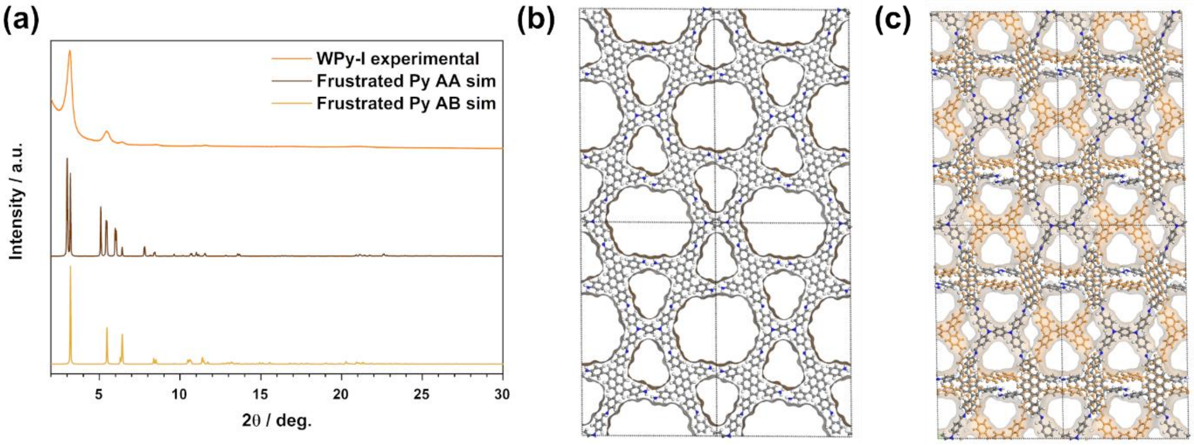

Figure S8. (**a**) Experimental powder XRD pattern of WPy-I COF (orange) and simulated PXRD patterns for eclipsed (brown) and staggered structure (beige) of frustrated bonded networks with Py– NH_2 serving as linear building block. Respective simulated crystal structures of (**b**) eclipsed structure and (**c**) staggered structure.

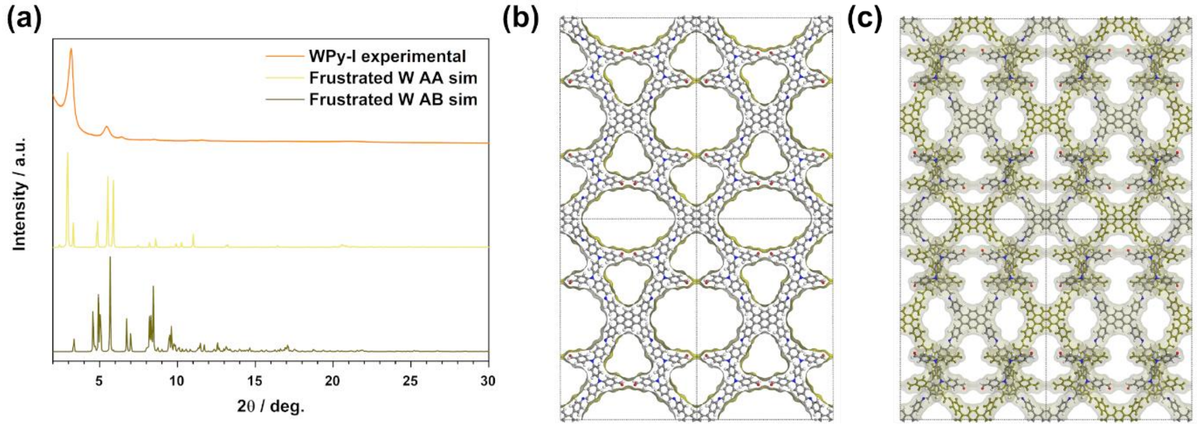

Figure S9. (**a**) Experimental powder XRD pattern of WPy-I COF (orange) and simulated PXRD patterns for eclipsed (dark yellow) and staggered structure (olive) of frustrated bonded networks with W–CHO serving as linear building block. Respective simulated crystal structures of (**b**) eclipsed structure and (**c**) staggered structure.

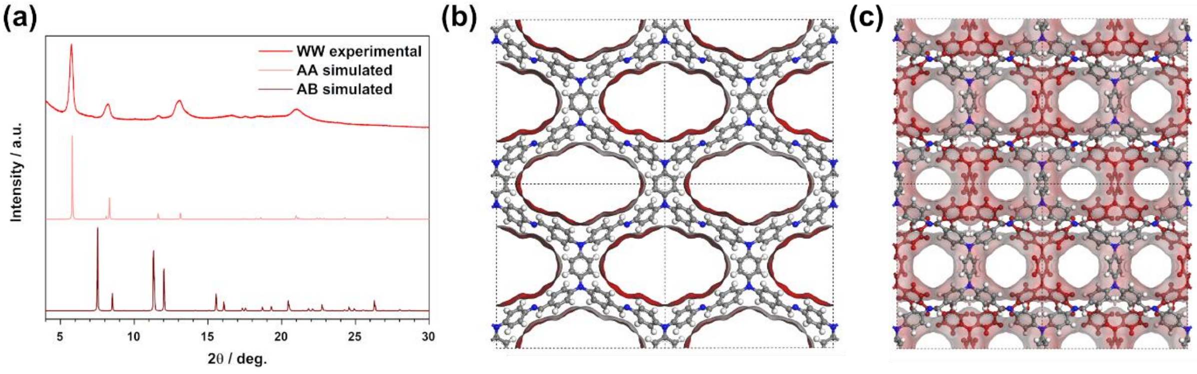

Figure S10. (**a**) Experimental powder XRD pattern of WW COF (red) and simulated PXRD patterns for eclipsed (light red) and staggered structure (wine). Respective simulated crystal structures of (**b**) eclipsed structure and (**c**) staggered structure.

S9. Nitrogen sorption

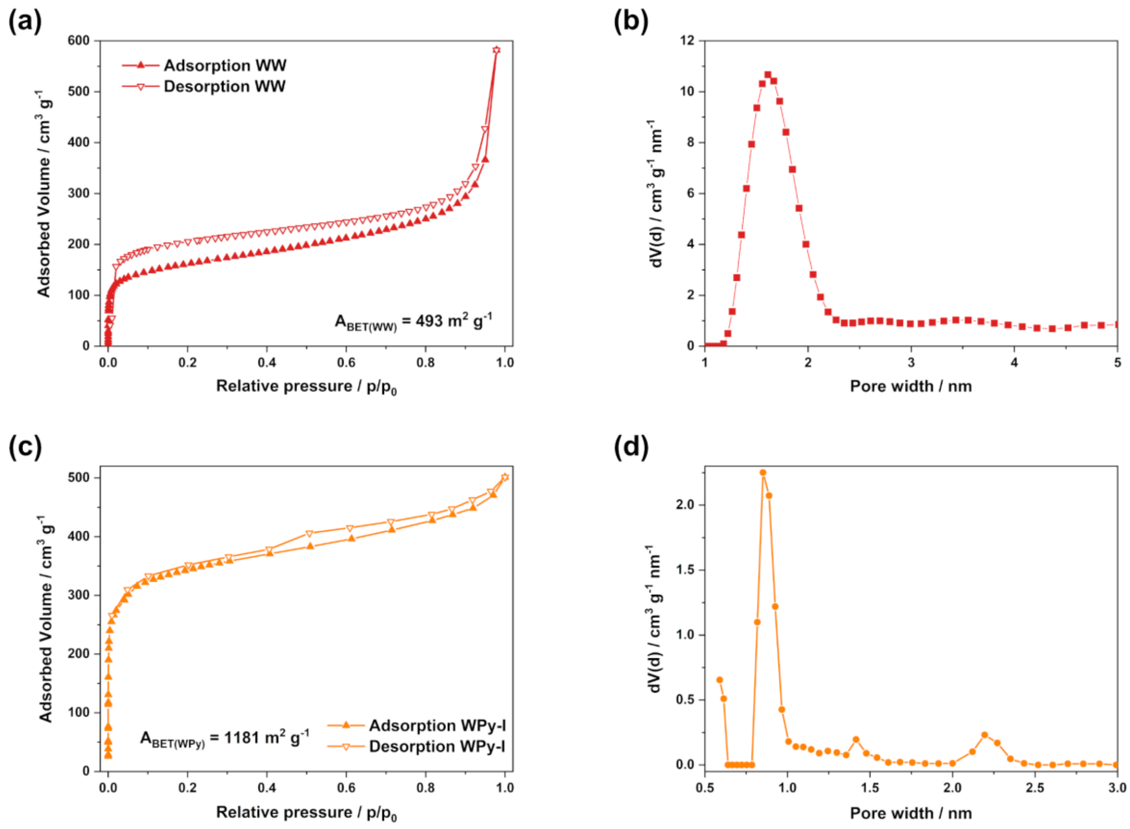

Figure S11. (**a,c**) Nitrogen adsorption and desorption isotherms and (**b,d**) the corresponding calculated pore size distributions of (**a,b**) WW and (**c,d**) WPy-I COF. Despite long equilibration times of five minutes per data point and a zero tolerance factor, the desorption equilibrium was always slightly higher than the adsorption equilibrium.

S10. Thermogravimetric Analysis

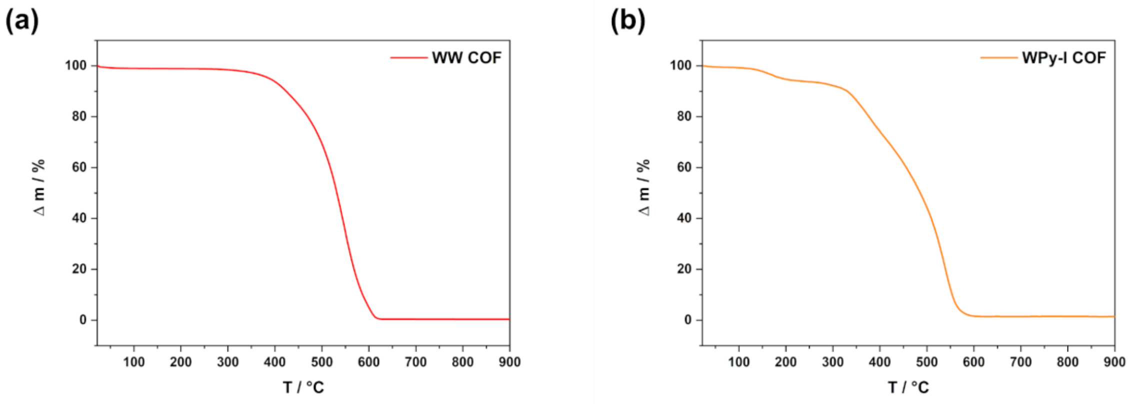

Figure S12. Thermogravimetric analysis of (**a**) WW and (**b**) WPy-I COFs. The minor weight losses up to 150 °C are attributed to solvent residues escaping from the solid.

S11. Scanning Electron Microscopy of COF bulk and films

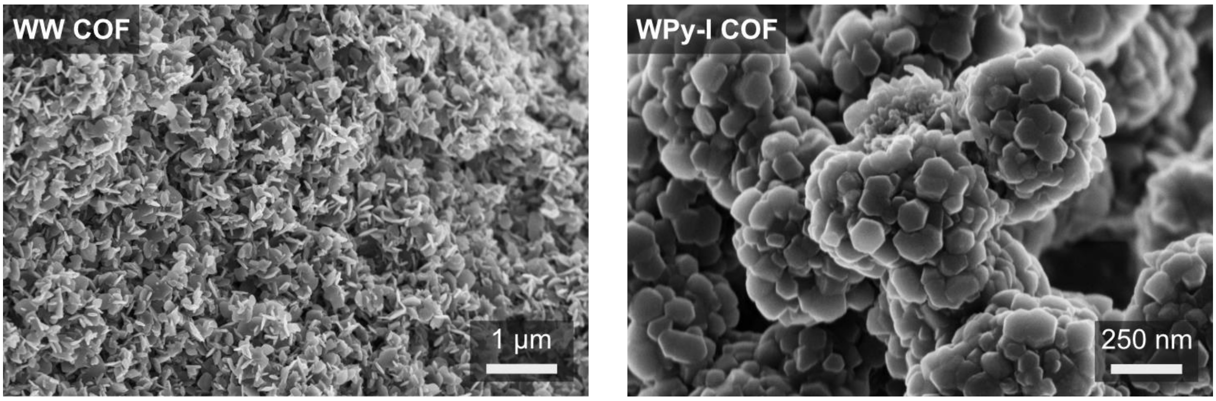

Figure S13. SEM image of WW COF showing the homogenous plate-like morphology (left) and SEM image of WPy-I COF showing hexagonal particle shapes.

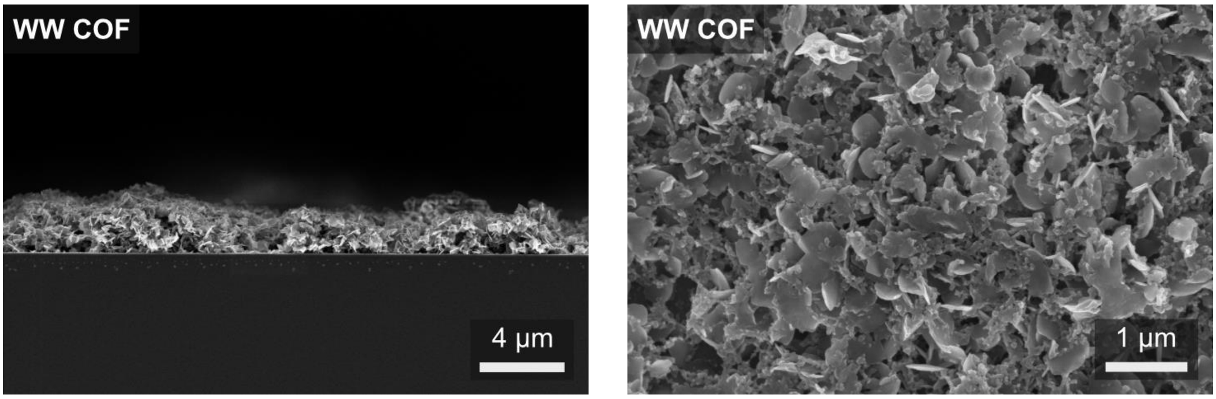

Figure S14. SEM top-view and cross-section images of WW films on glass.

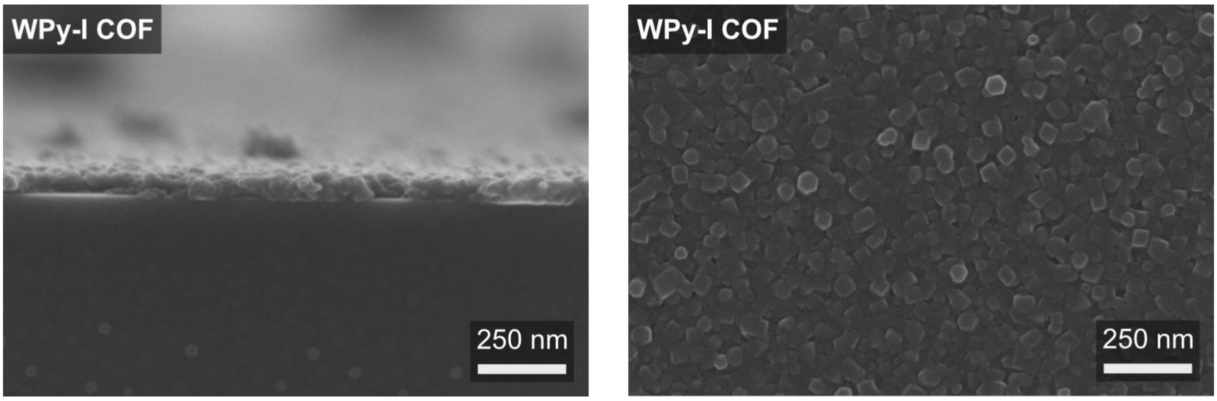

Figure S15. SEM top-view and cross-section images of WPy-I films on glass.

S12. Transmission Electron Microscopy

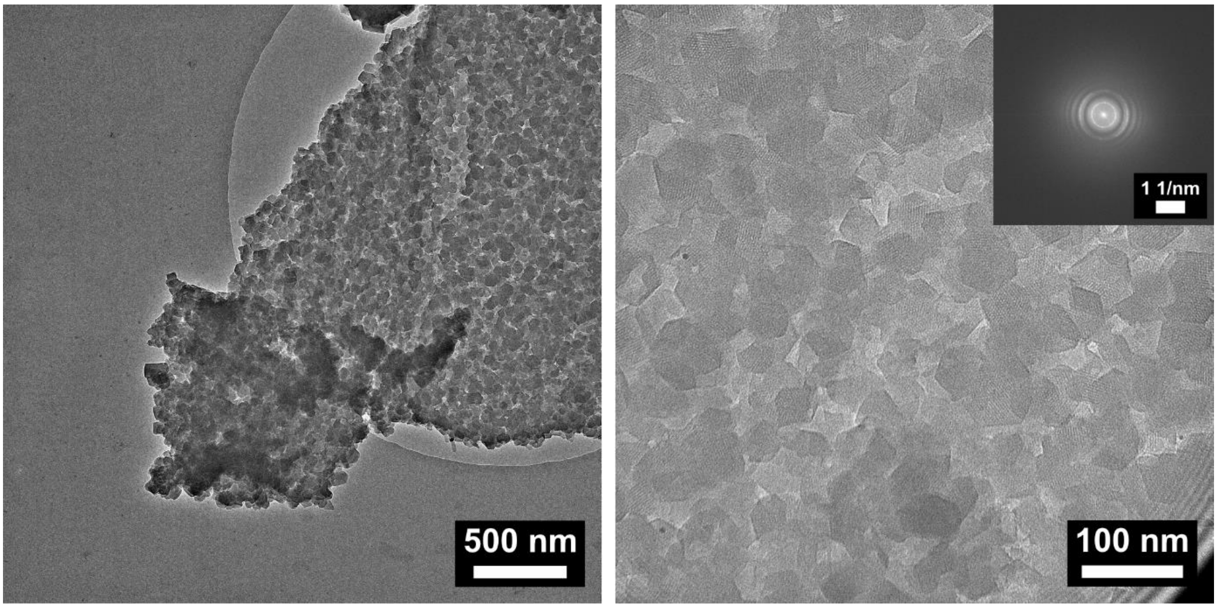

Figure S16. TEM images of WPy-I COF bulk material, shown at different magnifications. The Fourier transformation of the corresponding TEM image is shown as inset.

S13. Conductivity

Table S1. Average electrical conductivity values for WW and WPy-I COFs pressed pellets were measured using a van der Pauw four-probe setup at room temperature.

COF system	Conductivity (S cm^{-1})	Number of measurements n	Temperature (K)
**WW**	5.38 x 10^{-8} ± 0.48	10	292
**WPy-I**	3.74 x 10^{-8} ± 0.31	10	292

S14. Grazing incidence wide angle X-ray scattering

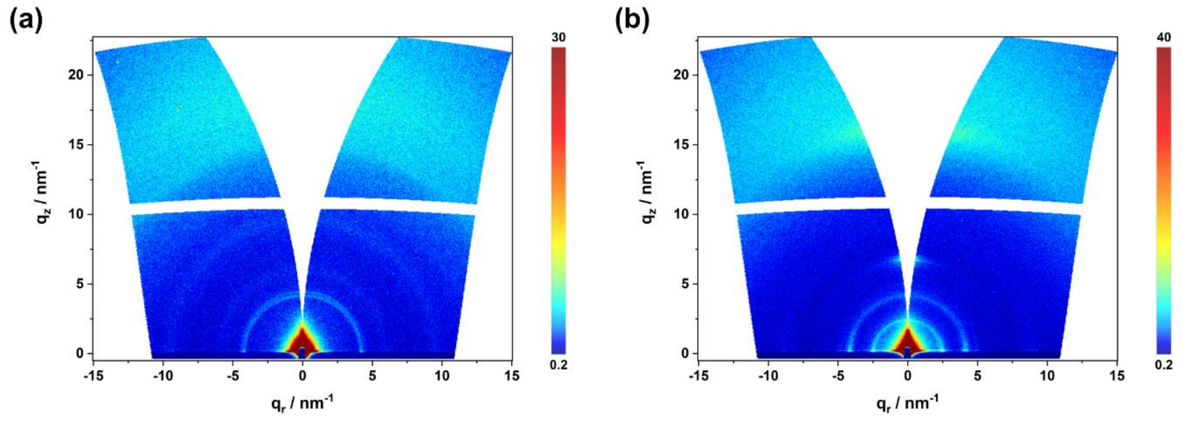

Figure S17. GIWAXS 2D pattern of **(a)** WW COF and **(b)** WPy-I COF on glass.

S15. Optical properties

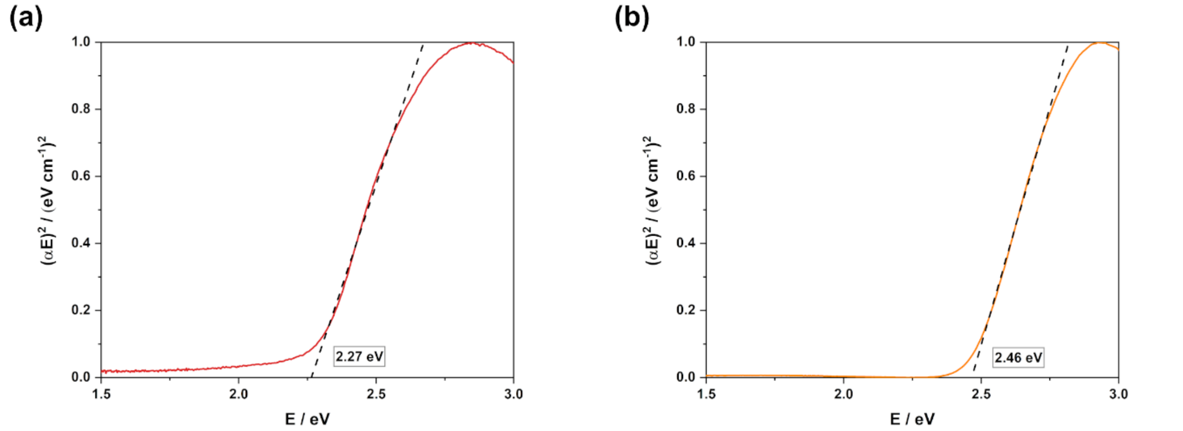

Figure S18. Tauc plots of the UV-Vis absorption data of **(a)** WW and **(b)** WPy-I COF.

S16. EDX mapping of the Ti/Ag/Al@SSMs

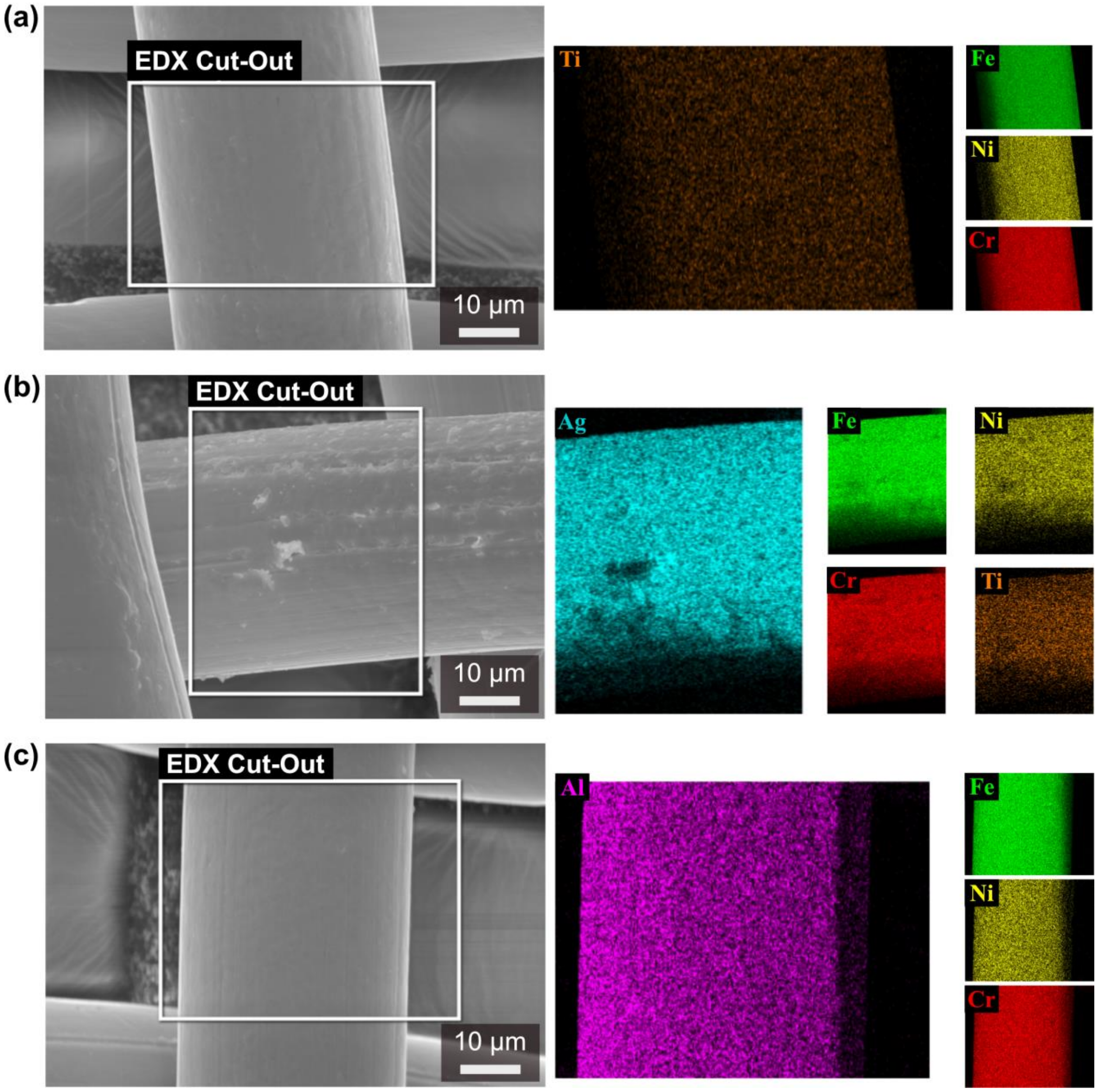

Figure S19. SEM images of the coated (**a**) Ti@SSM, (**b**) Ag@SSM and (**c**) Al@SSM with cut-outs containing color-coded EDX mapping of the elements titanium (orange), iron (green), nickel (yellow), chromium (red), silver (light blue) and aluminium (purple).

S17. Scanning Electron Microscopy of COF coated SSMs

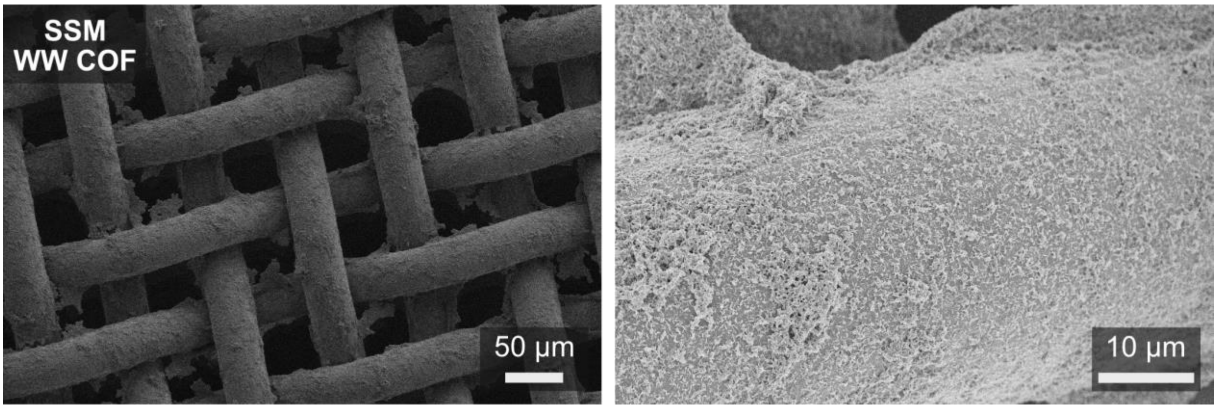

Figure S20. SEM images of the pure stainless steel mesh coated with WW COF.

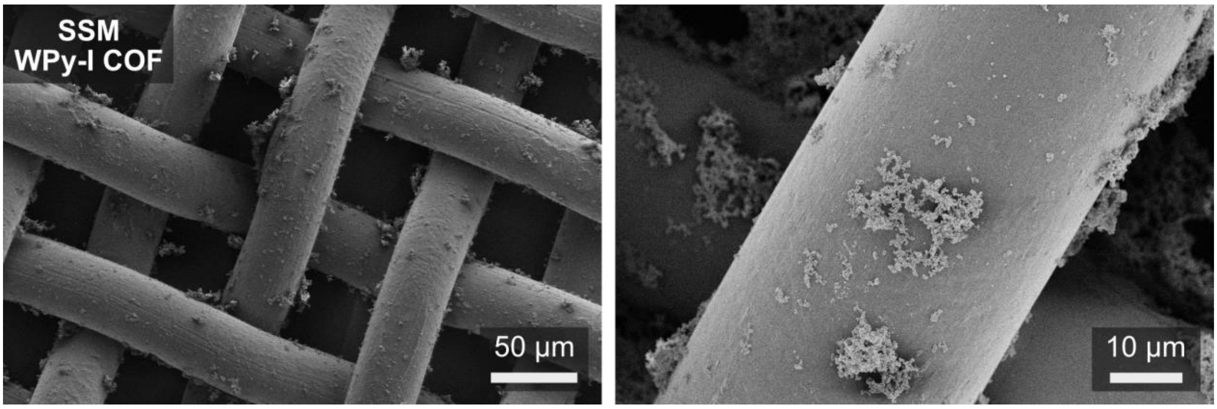

Figure S21. SEM images of the pure stainless steel mesh coated with WPy-I COF.

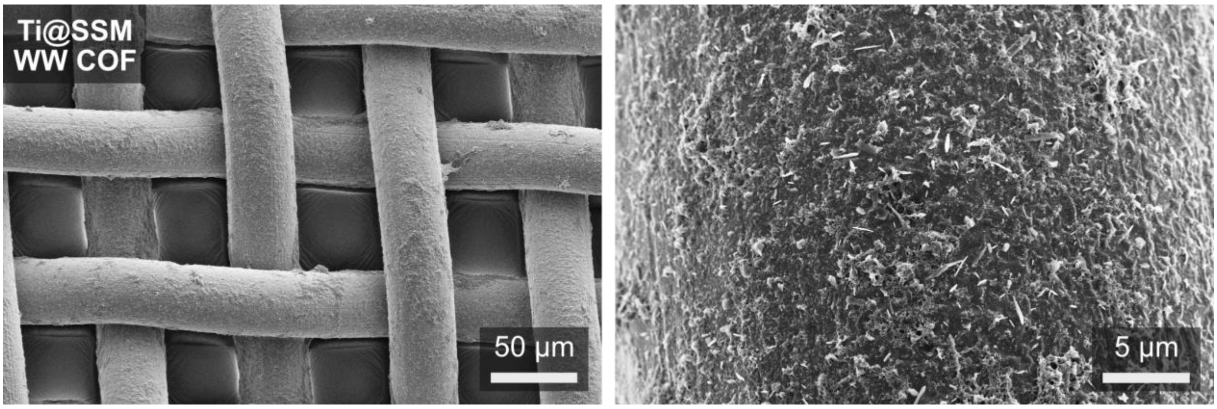

Figure S22. SEM images of the Ti@SSM coated with WW COF.

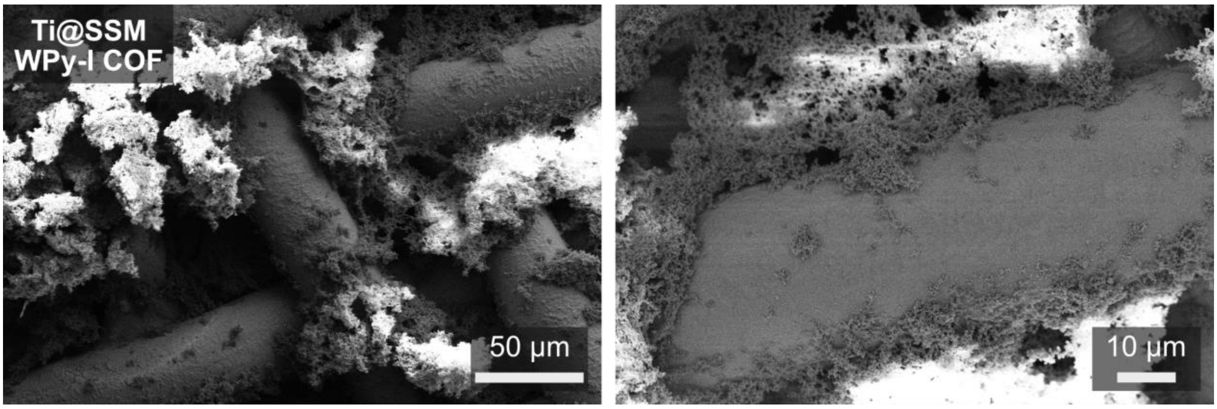

Figure S23. SEM images of the Ti@SSM coated with WPy-I COF.

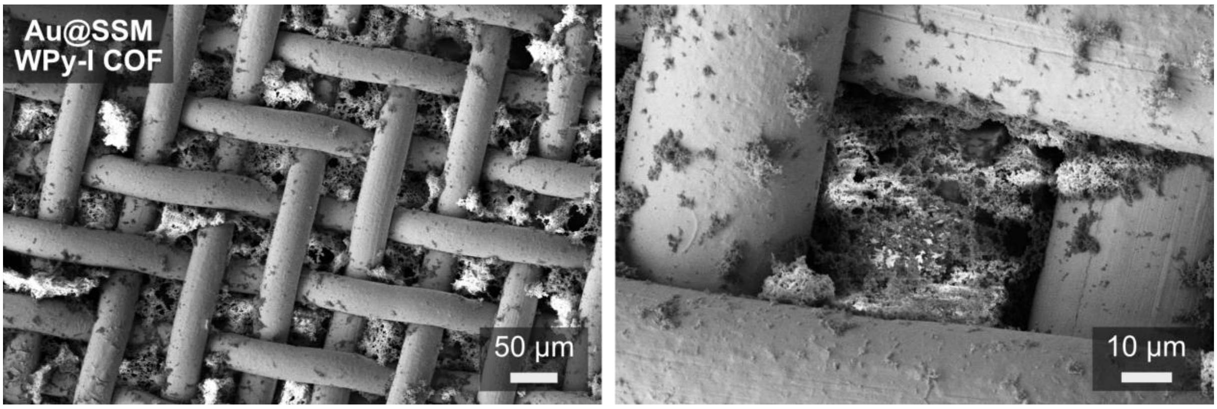

Figure S24. SEM images of the Au@SSM coated with WPy-I COF.

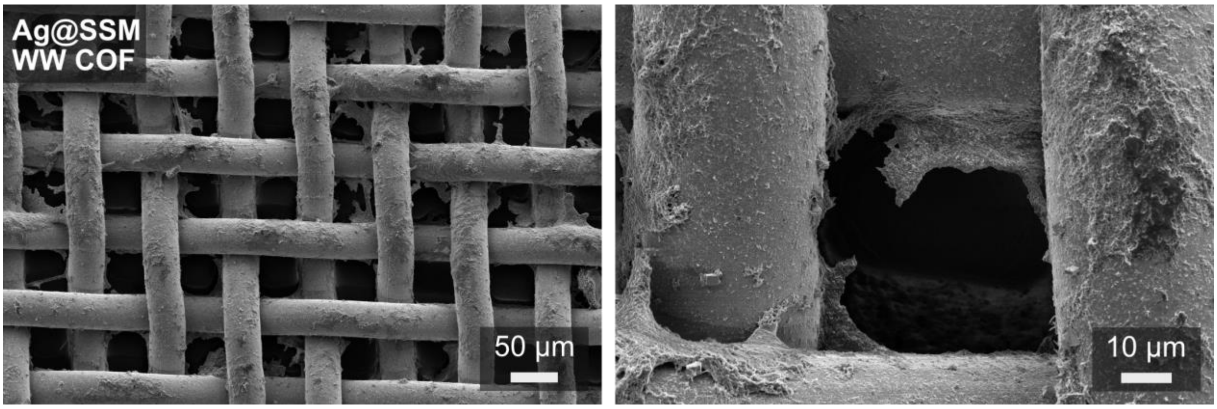

Figure S25. SEM images of the Ag@SSM coated with WW COF.

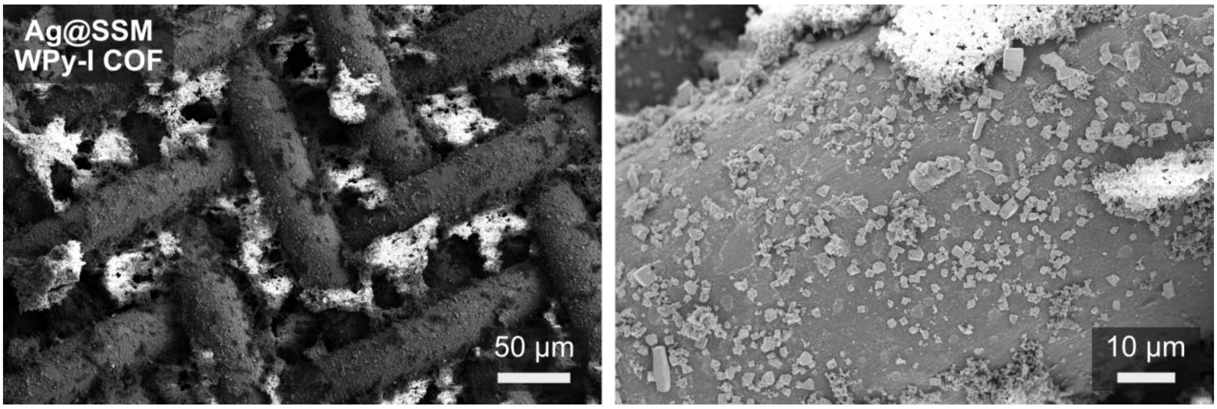

Figure S26. SEM images of the Ag@SSM coated with WPy-I COF.

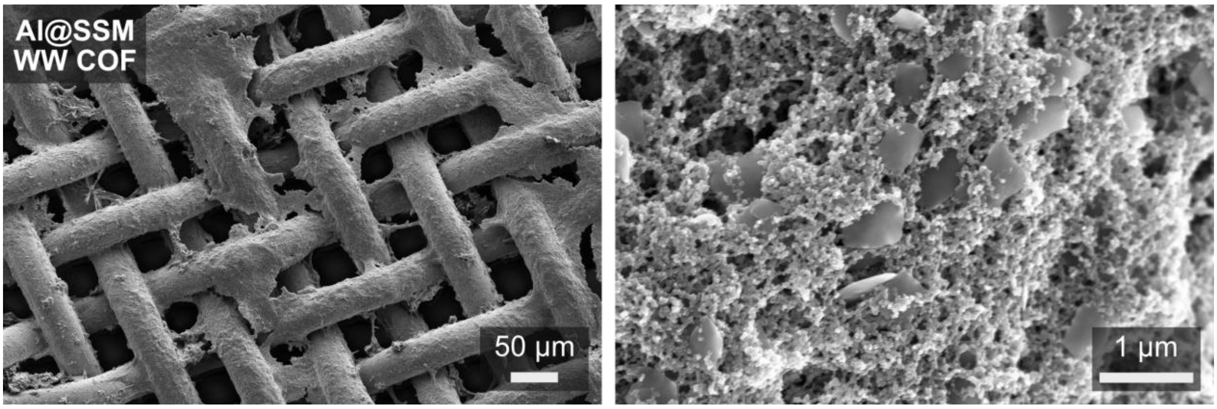

Figure S27. SEM images of the Al@SSM coated with WW COF.

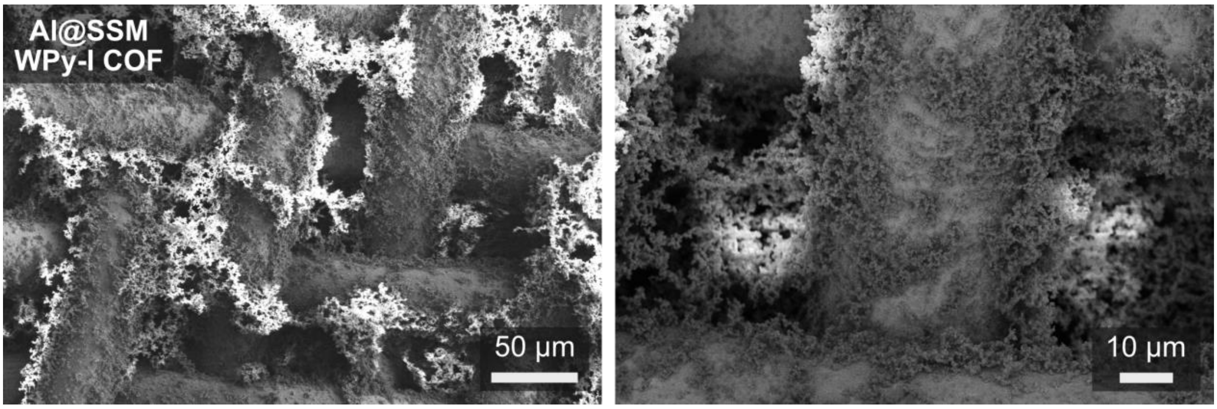

Figure S28. SEM images of the Al@SSM coated with WPy-I COF.

S18. XRD of the SSM active material

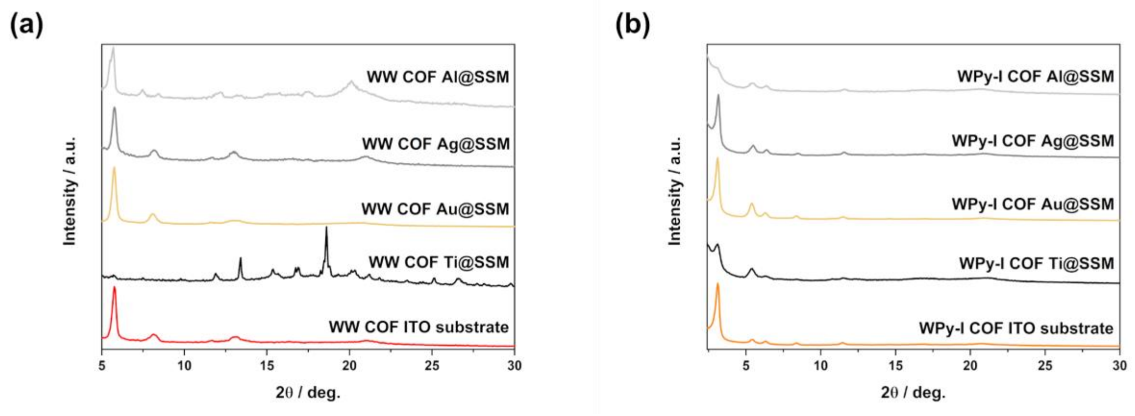

Figure S29. XRDs of (**a**) WW and (**b**) WPy-I COF obtained from scratched powder from the different SSMs and ITO substrates.

S19. Electrochemical characterization

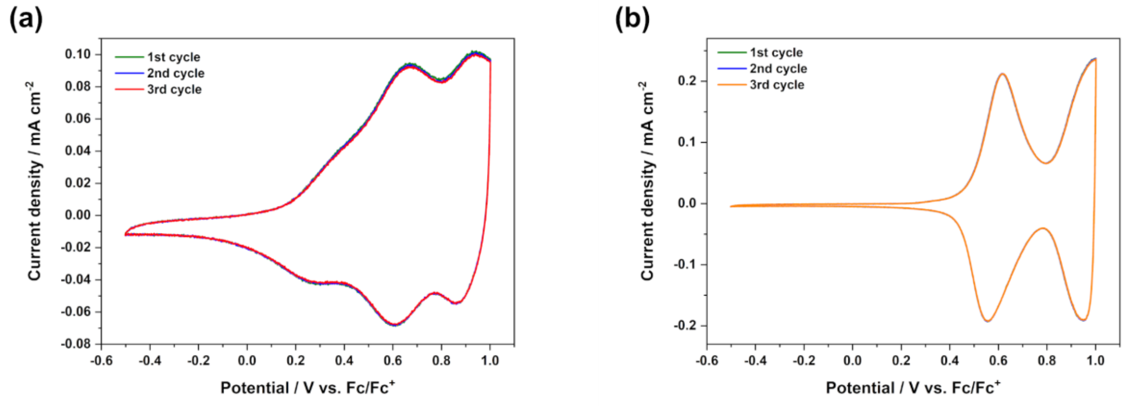

Figure S30. First CV cycles of (**a**) WW and (**b**) WPy-I COF films on ITO in a three-electrode cell with 1 M TBA PF_6 in acetonitrile serving as electrolyte at a scan rate of 100 mV s^{-1}.

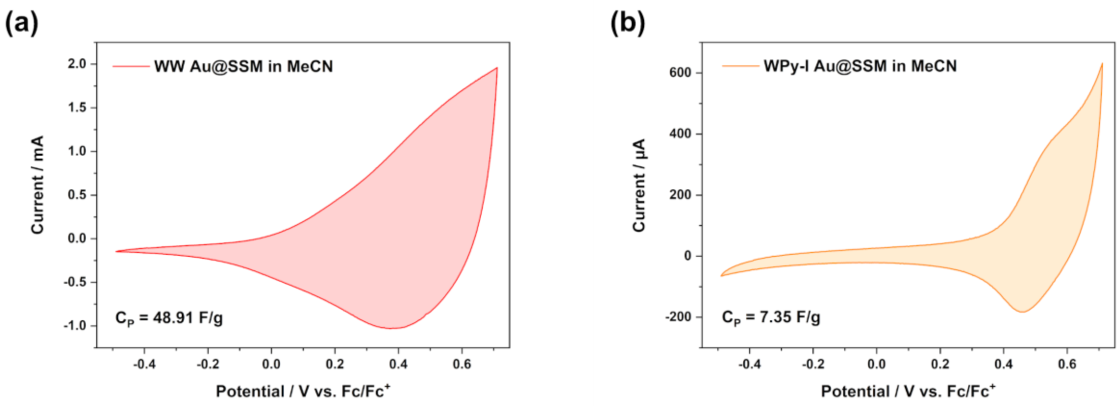

Figure S31. CV cycle of (**a**) WW and (**b**) WPy-I COF Au@SSMs in a three-electrode cell with 1 M TBA PF_6 in acetonitrile as electrolyte at a scan rate of 100 mV s^{-1}. The coloured area was used to calculate the specific capacitance C_P.

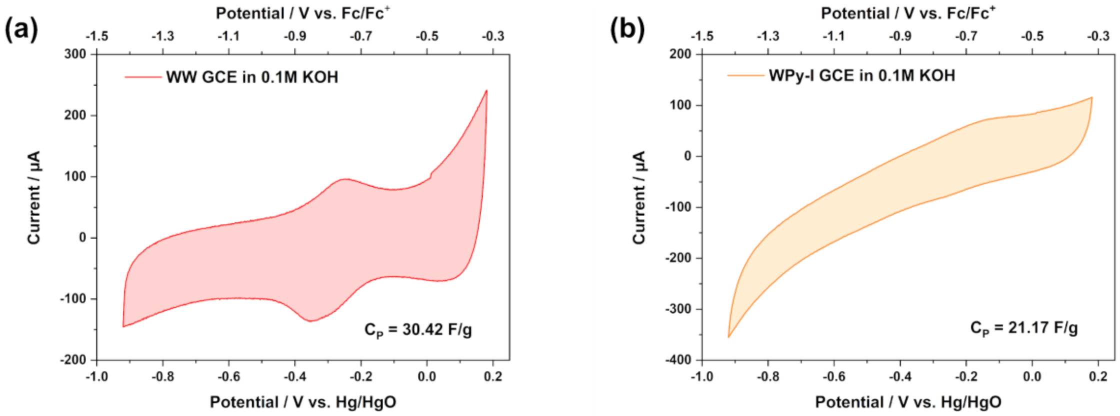

Figure S32. CV cycle of (**a**) WW and (**b**) WPy-I COF slurries (COF:super c65:PVDF=50:40:10) on a glassy carbon electrode in a three-electrode cell with 0.1 M KOH as electrolyte at a scan rate of 50 mV s^{-1}. The coloured area was used to calculate the specific capacitance C_P. The potential scale vs. Fc/Fc^+ is estimated for comparison.

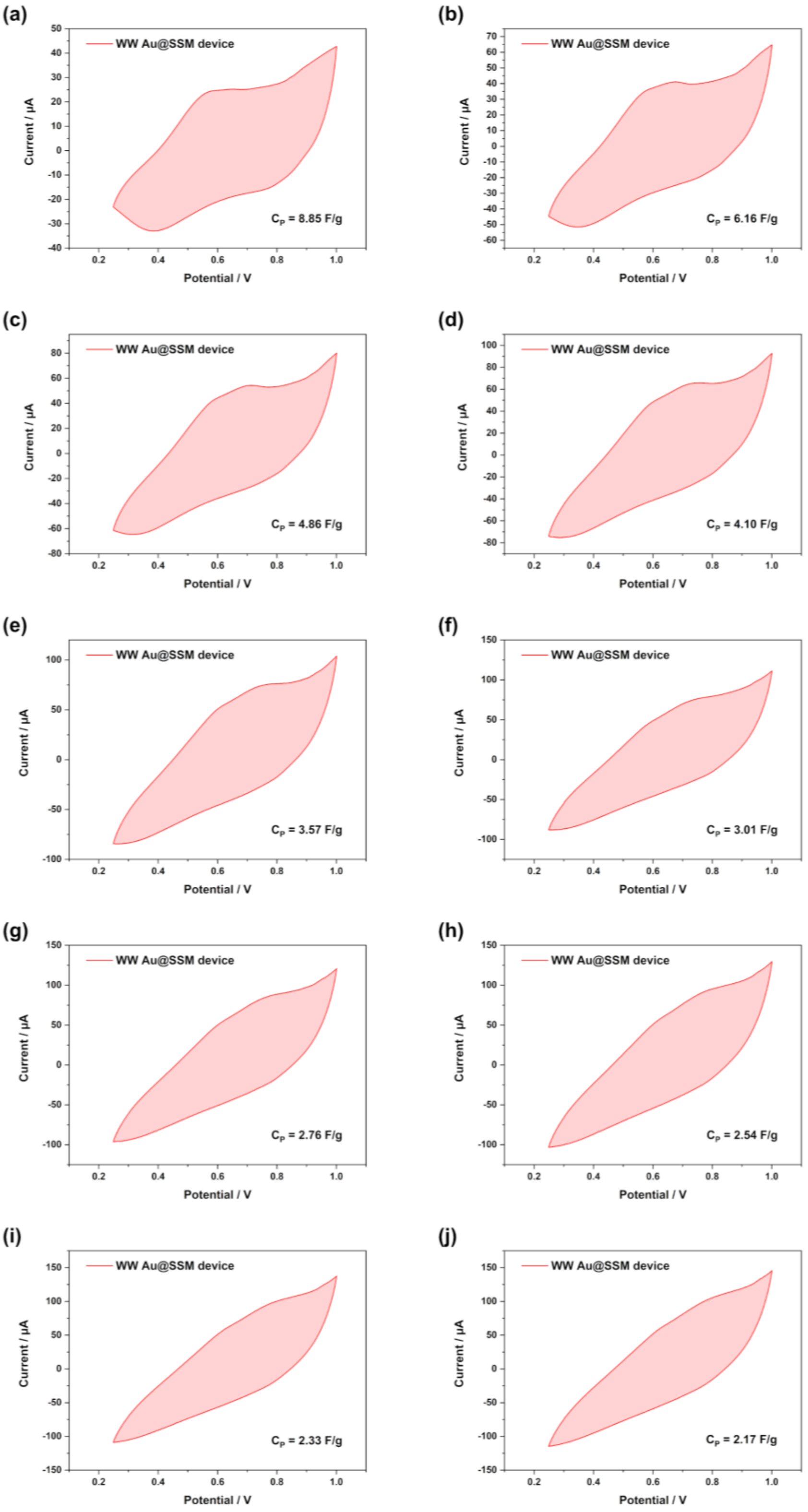

Figure S33. CV cycle of WW COF Au@SSM devices with 1-hexyl-3-methylimidazolium hexafluorophosphate as ionic liquid at a scan rate of (**a**) 10 mV s^{-1}, (**b**) 20 mV s^{-1}, (**c**) 30 mV s^{-1}, (**d**) 40 mV s^{-1}, (**e**) 50 mV s^{-1}, (**f**) 60 mV s^{-1}, (**g**) 70 mV s^{-1}, (**h**) 80 mV s^{-1}, (**i**) 90 mV s^{-1} and (**j**) 100 mV s^{-1}. The colored area was used to calculate the specific capacitance C_P.

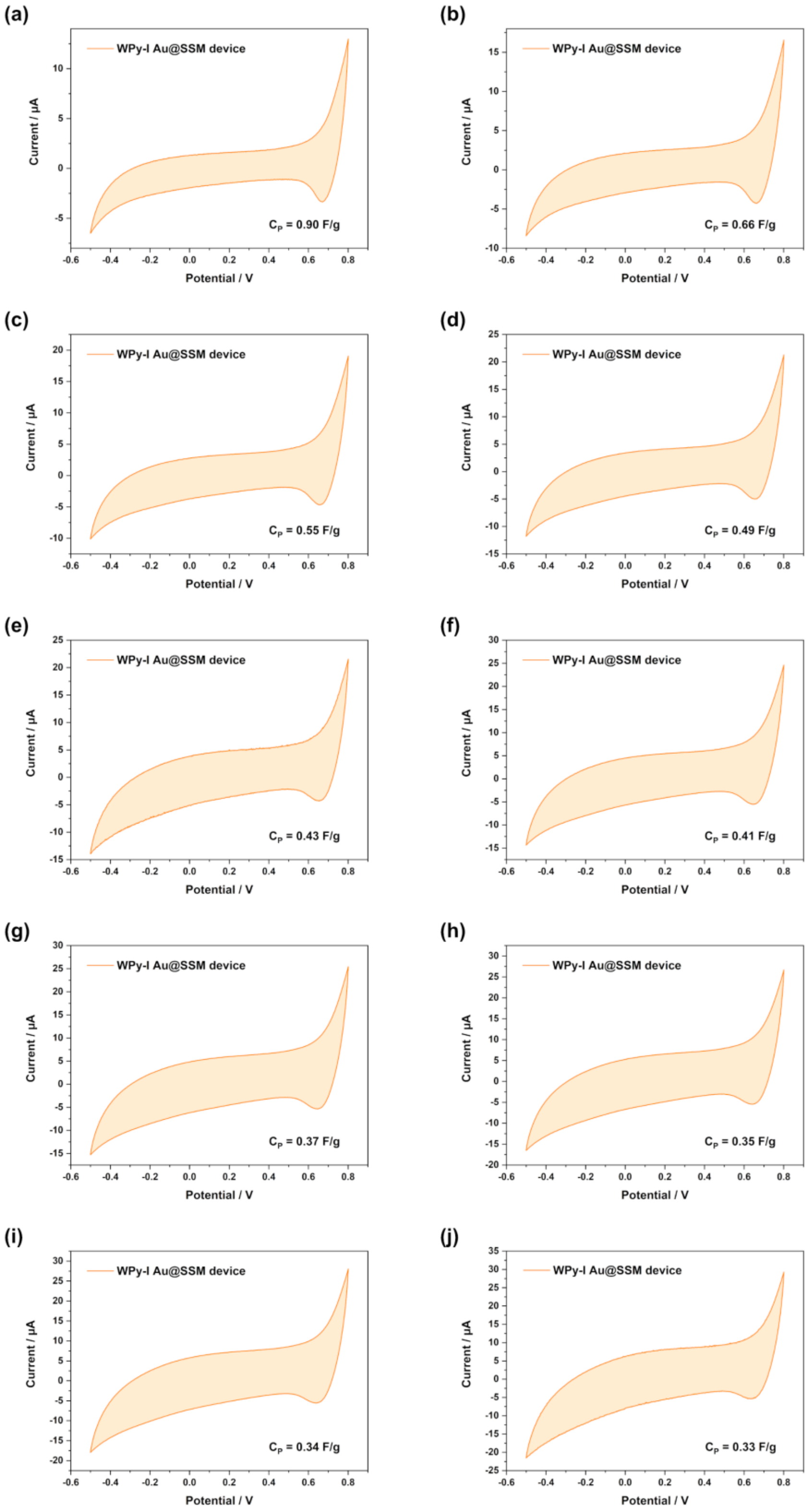

Figure S34. CV cycle of WPy-I COF Au@SSM devices with 1-hexyl-3-methylimidazolium hexafluorophosphate as ionic liquid at a scan rate of (**a**) 10 mV s^{-1}, (**b**) 20 mV s^{-1}, (**c**) 30 mV s^{-1}, (**d**) 40 mV s^{-1}, (**e**) 50 mV s^{-1}, (**f**) 60 mV s^{-1}, (**g**) 70 mV s^{-1}, (**h**) 80 mV s^{-1}, (**i**) 90 mV s^{-1} and (**j**) 100 mV s^{-1}. The colored area was used to calculate the specific capacitance C_P.

Table S2. Specific capacitance values of WW and WPy-I COF in different SSM devices and their corresponding potential ranges at different scan rates.

Device electrode material	Potential range / V	Scan rate / mV s^{-1}	Specific capacitance C_P / F g^{-1}
**WPy-I Au@SSM**	−0.5-0.8	10	0.90
**WPy-I Au@SSM**	−0.5-0.8	20	0.66
**WPy-I Au@SSM**	−0.5-0.8	30	0.55
**WPy-I Au@SSM**	−0.5-0.8	40	0.49
**WPy-I Au@SSM**	−0.5-0.8	50	0.43
**WPy-I Au@SSM**	−0.5-0.8	60	0.41
**WPy-I Au@SSM**	−0.5-0.8	70	0.37
**WPy-I Au@SSM**	−0.5-0.8	80	0.35
**WPy-I Au@SSM**	−0.5-0.8	90	0.34
**WPy-I Au@SSM**	−0.5-0.8	100	0.33
**WW Au@SSM**	0.25-1	10	8.85
**WW Au@SSM**	0.25-1	20	6.16
**WW Au@SSM**	0.25-1	30	4.86
**WW Au@SSM**	0.25-1	40	4.10
**WW Au@SSM**	0.25-1	50	3.57
**WW Au@SSM**	0.25-1	60	3.01
**WW Au@SSM**	0.25-1	70	2.76
**WW Au@SSM**	0.25-1	80	2.54
**WW Au@SSM**	0.25-1	90	2.33
**WW Au@SSM**	0.25-1	100	2.17

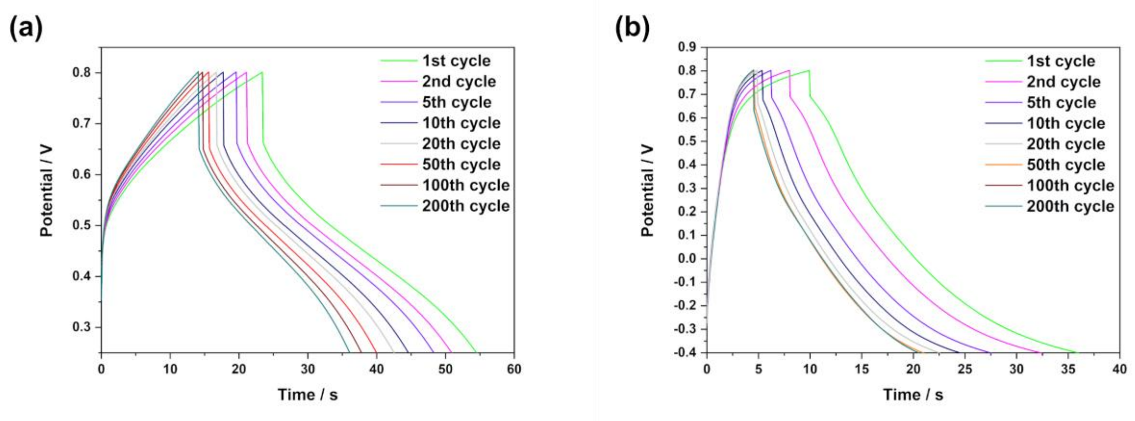

Figure S35. Galvanostatic charge-discharge profiles of (**a**) WW and (**b**) WPy-I COF Au@SSM devices at applied current density of 0.2 A g^{-1} until 200 cycles.

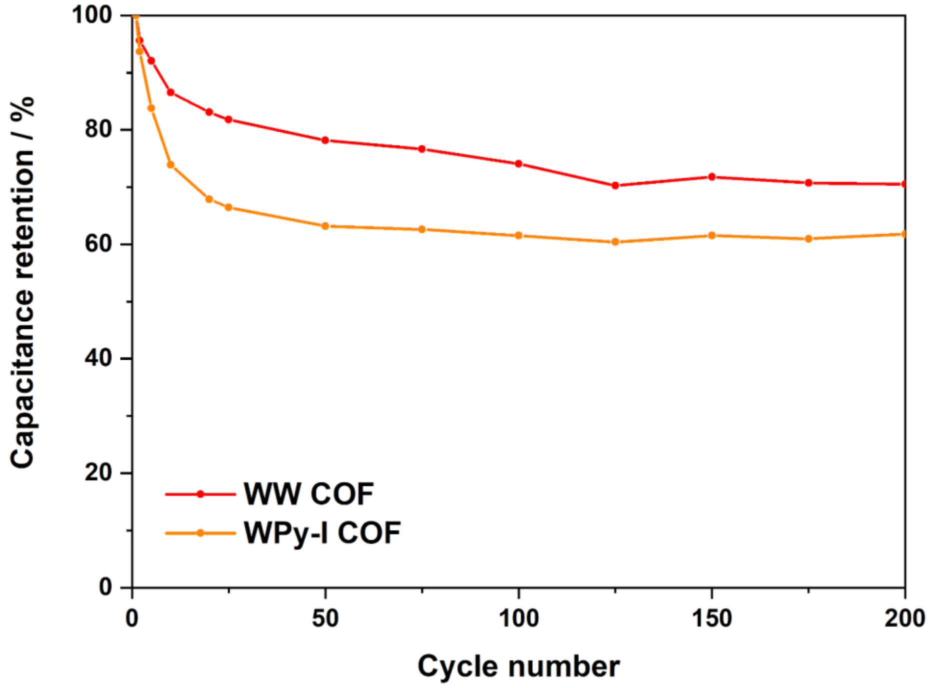

Figure S36. Cycling performance measured at a current density of 0.2 A g^{-1} for 200 cycles.

Table S3. Summary of reported electrochemical performance of COF-based supercapacitors.

COF	Carbon additive	Electrolyte	Specific capacitance C_P	Current density	Capacitance retention/ Cycles	Reference
**BFTB-PyTA**	45 wt % carbon black	1 M KOH	71 F g^{-1}	1 A g^{-1}	97.27% at 10 A/g/ 2000 cycles	[5]
**Car-TPT COF**	25 wt % super-P	0.5 M H_2SO_4	17.4 F g^{-1}	0.2 A g^{-1}	-	[6]
**[C_{60}]0.05-COF**	Fullerene C_{60} + 10 wt % acetylene black	1 M $NaSO_4$	63.1 F g^{-1}	0.7 A g^{-1}	90.6% at 4 A/g/ 5000 cycles	[7]
**DAAQ-TFP**	60 wt % carbon (12 F g^{-1})	1 M H_2SO_4	48 F g^{-1}	0.1 A g^{-1}	~81%/ 5000 cycles	[8]
**DAB-COF**	No additives, grown on carbon foam	1 M H_2SO_4	2.7 F g^{-1}	0.5 A g^{-1}	100% at 10 A/g/ 20000 cycles	[9]
**DHBD-Sb-COF**	10 wt % carbon black	1 M KOH	768 F g^{-1}	1 A g^{-1}	-	[10]
**Dq_1Da_1Tp COF**	graphite foil + carbon tape	1 M H_2SO_4	111 F g^{-1}	1.56 mA cm^{-2}	90%/ 7000 cycles	[11]
**Hex-Aza-COFs**	10 wt % conductive carbons + carbon fabric electrode	1 M KOH	600 F g^{-1}	1 A g^{-1}	89%/ 7500 cycles	[12]
**IISERPCOF-10**	25 wt % super-P carbon + carbon cloth	-	546 F g^{-1}	0.5 A g^{-1}	83%/ 10000 cycles	[13]
**PDC–MA–COF**	15 wt % acetylene black	6 M KOH	335 F g^{-1}	1 A g^{-1}	88%/ 20000 cycles	[14]
**PFM-COF1**	20 wt % carbon black	1 M H_2SO_4	394.28 F g^{-1}	0.5 A g^{-1}	81% at 3 A/g/ 2000 cycles	[15]
**Phos-COF-1**	10 wt % carbon black	3 M Na_2SO_4	100 F g^{-1}	1 A g^{-1}	90% at 4 A/g/ 5000 cycles	[16]

COF	Carbon additive	Electrolyte	Specific capacitance C_P	Current density	Capacitance retention/ Cycles	Reference
**Sb-COF**	20 wt % carbon black	1 M KOH	260 F g^{-1}	2 A g^{-1}	80%/ 100000 cycles	[17]
**"TAPA-TPT COF"**	COF was carbonized at 800 °C	1 M H_2SO_4	205 F g^{-1}	0.5 A g^{-1}	95% at 15 A/g/ 5000 cycles	[18]
**TaPa-Py COF**	25 wt % carbon black + carbon paper	1 M H_2SO_4	209 F g^{-1}	0.5 A g^{-1}	92%/ 6000 cycles	[19]
**TFP-NDA-COF**	"ink", no further details	1 M H_2SO_4	348 F g^{-1}	0.5 A g^{-1}	75%/ 8000 cycles	[20]
**TPA-COF-1**	25 wt % super-P	0.5 M H_2SO_4	51.3 F g^{-1}	0.2 A g^{-1}	-	[21]
**TpOMe-DAQ**	No additives	3 M H_2SO_4	169 F g^{-1}	3.3 mA cm^{-2}	100%/ 100000 cycles	[22]
**TpPa-$(OH)_2$**	10 wt % carbon black	1 M phosphate buffer (pH = 7.2)	416 F g^{-1}	0.5 A g^{-1}	88%/ 10000 cycles	[23]
**TPPDA-TPPyr**	45 wt % carbon black	1 M KOH	188.7 F g^{-1}	2 A g^{-1}	85.6%/ 5000 cycles	[24]
**TPPDA-TPTPE**	45 wt % carbon black	1 M KOH	237.1 F g^{-1}	2 A g^{-1}	86.2%/ 5000 cycles	[24]
**TPT-DAHQ COF**	45 wt % carbon black	1 M KOH	256 F g^{-1}	0.5 A g^{-1}	98%/ 1850 cycles	[25]
**TTT-DHTD COF**	30 wt % carbon (Ketjen Black)	1 M KOH	273.3 F g^{-1}	0.5 A g^{-1}	0.025% decay per cycle/ 2000 cycles	[26]
**250-HADQ COF**	10 wt % super P carbon black	$EMIMBF_4$ + 1 M H_2SO_4	516.4 F g^{-1}	0.5 A g^{-1}	81% at 2 A/g/ 1000000 cycles	[27]
**4KT-Tp COF**	56 wt% conductive carbon black	1.0 M H_2SO_4	583 F g^{-1}	0.2 A g^{-1}	92% at 5 A/g/ 20000 cycles	[28]

S20. Electrochemical reference measurements

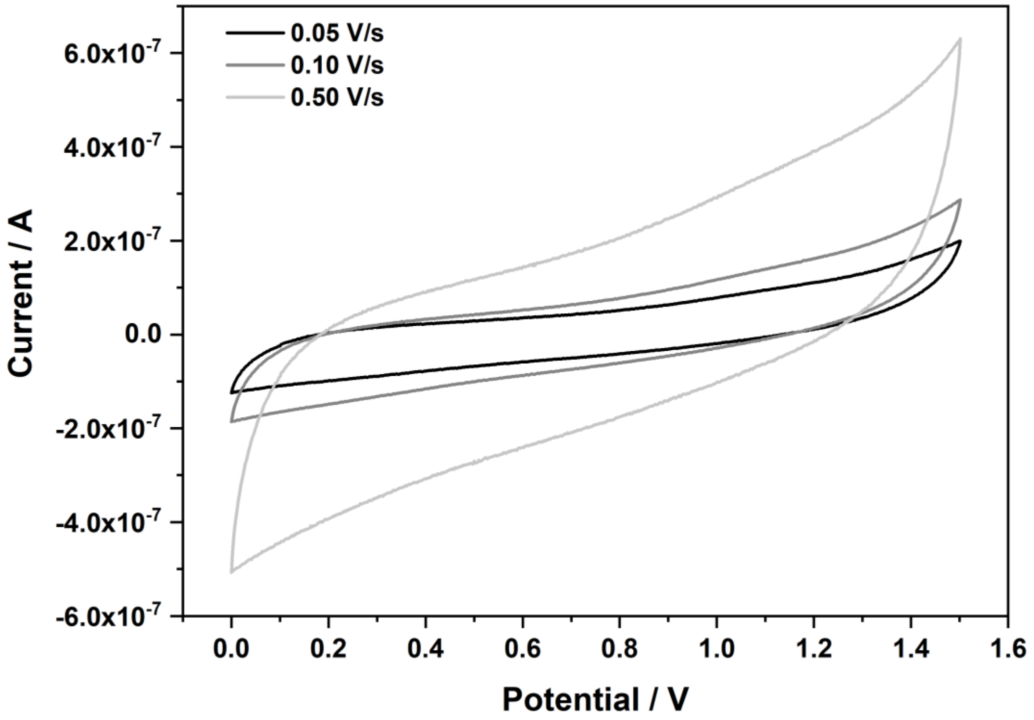

Figure S37. Reference CVs of the SSM device with ionic liquid and without active material.

Unit cell parameters (*P2/m*) and atomic coordinates for WW COF

**a = 22.0982 Å, b = 21.2518 Å, c = 4.2854 Å**			
**α = 90.0000° β = 99.0018°, ɣ = 90.0000°**			
**Atom**	**a/x**	**b/y**	**c/z**
C1	0.55338	0.5319	0.2951
C2	0.55841	0.65898	0.45357
C3	0.57961	0.70776	0.26198
C4	0.63852	0.74121	0.21896
C5	0.67842	0.72698	0.36692
C6	0.6582	0.67736	0.55421
C7	0.599	0.64421	0.59755
C8	0.73589	0.76251	0.32687
N9	0.77521	0.75239	0.46747
C10	0.83051	0.78247	0.46098
C11	0.85787	0.83518	0.30174
C12	0.91524	0.86369	0.30953
C13	0.94627	0.8421	0.48244
C14	0.91868	0.78885	0.6392
C15	0.86204	0.75939	0.62748
C16	1.05739	0.96811	0.31962
H17	0.59283	0.55423	0.14331
H18	0.55179	0.71912	0.15203
H19	0.65189	0.7762	0.07811
H20	0.68628	0.66528	0.66245
H21	0.58495	0.60922	0.73736
H22	0.74766	0.79749	0.18707
H23	0.83661	0.85339	0.17845
H24	0.9344	0.90114	0.18815
H25	0.93952	0.77149	0.7664
H26	0.8433	0.72099	0.74319
H27	1.09977	0.94566	0.18653
N28	0.5	0.37255	0.5
C29	0.5	0.43481	0.5
N30	1	0.12747	0.5
C31	1	0.06509	0.5

Unit cell parameters (*P1*) and atomic coordinates for WPy-I COF				Unit cell parameters (*P2/m*) and atomic coordinates for WPy-II COF			
**a = 32.2957 Å, b = 55.1110 Å, c = 8.7677 Å**				**a = 46.5254 Å, b = 46.9146 Å, c = 4.6815 Å**			
**α = 92.5232° β = 89.1079°, ɣ = 90.9651°**				**α = 90.0000° β = 82.3909°, ɣ = 90.0000°**			
**Atom**	**a/x**	**b/y**	**c/z**	**Atom**	**a/x**	**b/y**	**c/z**
C1	0.67665	0.76528	0.54919	C1	0.28354	0.7745	-1.01374
C2	0.69654	0.74313	0.51631	C2	0.28497	0.80391	-0.93882
C3	0.67639	0.72056	0.53952	C3	0.30832	0.75739	-1.05988
C4	0.79678	0.7223	0.34451	H4	0.33056	0.767	-1.04433
C5	0.75825	0.72152	0.42111	C5	0.3121	0.81845	-0.88963
C6	0.73949	0.6992	0.4607	C6	0.3288	0.80803	-0.68579
C7	0.69821	0.69902	0.51281	C7	0.35414	0.82211	-0.63945
C8	0.73733	0.74355	0.45421	C8	0.36287	0.84719	-0.78942
H9	0.64543	0.76591	0.59493	C9	0.34573	0.85802	-0.98946
H10	0.81177	0.70582	0.3031	C10	0.32059	0.84378	-1.03795
H11	0.6831	0.68196	0.53864	N11	0.38859	0.86123	-0.7248
C12	0.67951	0.09124	0.49797	H12	0.3217	0.78801	-0.55746
H13	0.70177	0.09238	0.58891	H13	0.36812	0.81328	-0.4776
N14	0.83275	0.61146	0.46137	H14	0.35241	0.87848	-1.11208
C15	0.81826	0.58969	0.48109	H15	0.30682	0.85289	-1.20029
C16	0.84545	0.56842	0.46669	C16	0.1242	0.89659	-0.9709
C17	0.88429	0.5696	0.3924	C17	0.10649	0.92209	-0.89375
C18	0.90843	0.54875	0.37219	C18	0.08341	0.92937	-1.04085
C19	0.89442	0.52654	0.4279	C19	0.06849	0.9552	-0.97813
C20	0.85609	0.52568	0.50595	C20	0.07605	0.97353	-0.76353
C21	0.83169	0.54632	0.52382	C21	0.09857	0.96551	-0.61265
C22	0.96247	0.50457	0.40739	C22	0.11387	0.94036	-0.67945
N23	0.9178	0.5046	0.39945	H23	0.07661	0.91442	-1.21183
C24	0.02656	0.51899	0.52545	H24	0.04999	0.96146	-1.10254
C25	0.02903	0.48872	0.31772	H25	0.1047	0.9796	-0.43171
C26	0.98331	0.51939	0.51645	H26	0.13267	0.93477	-0.55636
C27	0.98576	0.48917	0.30841	H27	0.12564	0.88735	-1.19757
H28	0.89531	0.58652	0.34691	C28	0.28017	0.71548	-1.15859
H29	0.93758	0.54973	0.31021	C29	0.27818	0.68585	-1.21868
H30	0.8449	0.50882	0.55145	C30	0.30661	0.72843	-1.12545

H31	0.80198	0.54504	0.58225	H31	0.32707	0.71479	-1.15318
H32	0.04203	0.5305	0.61072	C32	0.30253	0.66599	-1.20517
H33	0.04646	0.47702	0.23897	C33	0.32798	0.66781	-1.39756
H34	0.96593	0.53104	0.59562	C34	0.35149	0.6503	-1.35931
H35	0.97026	0.47768	0.22316	C35	0.34954	0.63078	-1.13239
H36	0.78599	0.58689	0.51268	C36	0.32355	0.62797	-0.95075
C37	0.25513	0.3355	0.47536	C37	0.30033	0.64556	-0.98547
C38	0.13834	0.22889	0.66993	N38	0.37527	0.61308	-1.08362
C39	0.27259	0.35816	0.52036	H39	0.32956	0.6835	-1.58493
C40	0.25082	0.37954	0.49816	H40	0.37251	0.65189	-1.51419
C41	0.21123	0.37833	0.43328	H41	0.32132	0.61118	-0.77308
C42	0.19461	0.35589	0.38511	H42	0.27925	0.64323	-0.83257
C43	0.21686	0.33481	0.40182	C43	0.19856	0.67313	-1.13319
C44	0.11011	0.2404	0.57796	C44	0.17533	0.67862	-1.28522
C45	0.06772	0.23962	0.61289	C45	0.14905	0.66448	-1.21008
C46	0.05308	0.22709	0.73855	C46	0.14583	0.64417	-0.98897
C47	0.0811	0.2157	0.83098	C47	0.16985	0.63706	-0.85324
C48	0.12355	0.21657	0.797	C48	0.19578	0.65166	-0.9225
C49	0.81669	0.41866	0.33143	N49	0.11804	0.63136	-0.90979
C50	0.84471	0.44009	0.34314	H50	0.17787	0.69461	-1.4702
N51	0.00974	0.22573	0.77142	H51	0.12979	0.66939	-1.32927
C52	0.83955	0.45934	0.24607	H52	0.16814	0.61928	-0.68585
C53	0.86465	0.48021	0.26108	H53	0.21499	0.64598	-0.80545
C54	0.89543	0.48205	0.37218	C54	0.37505	0.59748	-0.84855
C55	0.9016	0.46254	0.4656	H55	0.35749	0.60093	-0.65764
C56	0.87636	0.44172	0.45168	C56	0.22763	0.71943	-1.13253
H57	0.30238	0.35923	0.57538	C57	0.22595	0.68952	-1.18473
H58	0.26398	0.39684	0.53665	C58	0.20287	0.73659	-1.0824
H59	0.1644	0.35491	0.33338	H59	0.18046	0.72677	-1.08311
H60	0.20427	0.31769	0.35851	C60	0.20787	0.82724	-0.90613
H61	0.12095	0.2495	0.47759	C61	0.18585	0.82324	-0.67819
H62	0.0461	0.24844	0.54125	C62	0.16217	0.84167	-0.64492
H63	0.06995	0.20609	0.92864	C63	0.16022	0.864	-0.83919
H64	0.14487	0.2076	0.86913	C64	0.18228	0.86812	-1.06606
H65	0.81551	0.45843	0.16119	C65	0.20608	0.84986	-1.09891
H66	0.85972	0.49528	0.18884	N66	0.13803	0.88486	-0.77939
H67	0.92502	0.46393	0.55253	H67	0.18726	0.80497	-0.52008

H68	0.88105	0.427	0.52706	H68	0.14411	0.83863	-0.45771
H69	0.79542	0.41708	0.23742	H69	0.18085	0.88637	-1.22422
C70	0.63237	0.71862	0.58889	H70	0.2242	0.85342	-1.28446
C71	0.76313	0.67621	0.46045	C71	0.23132	0.7788	-1.00563
C72	0.80255	0.67601	0.52638	C72	0.23323	0.80826	-0.94059
C73	0.82513	0.65461	0.52565	C73	0.20477	0.7658	-1.03073
C74	0.80795	0.63282	0.46706	H74	0.18438	0.77954	-1.00818
C75	0.76783	0.63264	0.40731	C75	0.25983	0.82035	-0.90803
C76	0.74578	0.65423	0.40233	C76	0.25659	0.76193	-1.04659
C77	0.60332	0.70708	0.49164	H77	0.26095	0.84402	-0.85549
C78	0.56167	0.7053	0.53693	C78	0.25131	0.67358	-1.23753
C79	0.54867	0.71501	0.67934	C79	0.25485	0.73237	-1.11838
C80	0.57759	0.72636	0.77712	H80	0.24982	0.65011	-1.29701
C81	0.61928	0.728	0.73258	C81	0.48569	0.52587	0.47452
N82	0.19805	0.60466	0.4134	C82	0.57387	0.52622	0.64911
C83	0.19819	0.58805	0.51366	C83	0.56432	0.5431	0.88872
C84	0.16929	0.56711	0.49928	C84	0.57962	0.56758	0.95174
N85	0.5061	0.71346	0.72426	C85	0.6054	0.57469	0.77922
C86	0.13882	0.56585	0.3861	C86	0.61436	0.55834	0.53546
C87	0.11267	0.54547	0.36995	C87	0.5986	0.53449	0.46936
C88	0.1168	0.52608	0.46569	H88	0.47412	0.54705	0.45442
C89	0.14624	0.52761	0.58167	H89	0.54378	0.53707	1.03694
C90	0.17224	0.54804	0.59893	H90	0.57098	0.58155	1.14322
C91	0.04985	0.50363	0.42639	H91	0.63485	0.56438	0.38647
N92	0.09449	0.50368	0.43428	H92	0.60618	0.52176	0.26588
H93	0.8159	0.69251	0.57781	C93	0.98524	1.02586	0.54481
H94	0.85591	0.65479	0.57274	H94	0.97323	1.04704	0.57639
H95	0.75422	0.61598	0.35994	C95	0.8898	1.61626	0.67995
H96	0.71547	0.65385	0.35115	C96	0.91879	1.60362	0.62404
H97	0.61291	0.69971	0.38037	C97	0.92405	1.58159	0.42095
H98	0.53935	0.69653	0.46112	C98	0.95095	1.56825	0.37566
H99	0.56771	0.73404	0.88707	C99	0.97314	1.57699	0.52841
H100	0.64129	0.73701	0.80815	C100	0.96828	1.6	0.72015
H101	0.13582	0.58048	0.30875	C101	0.94122	1.61301	0.77098
H102	0.09031	0.54424	0.27925	C102	0.9781	1.515	0.66557
H103	0.14962	0.51256	0.65537	H103	0.87362	1.61294	0.52025
H104	0.1953	0.5487	0.68738	H104	0.9062	1.57451	0.29165

H105	0.21837	0.58958	0.61097	H105	0.95489	1.55011	0.21316
C106	0.26365	0.67188	0.42799	H106	0.98664	1.60826	0.83657
C107	0.22981	0.66759	0.33197	H107	0.93734	1.63128	0.93203
C108	0.20963	0.6451	0.32516	H108	0.95981	1.52673	0.80381
C109	0.22279	0.62647	0.4142	C109	0.40096	1.11665	-0.85692
C110	0.25851	0.63006	0.50301	C110	0.42695	1.10358	-0.76568
C111	0.2788	0.65277	0.51026	C111	0.44163	1.1162	-0.55591
N112	0.18586	0.39923	0.42481	C112	0.46607	1.1029	-0.47307
C113	0.19926	0.42079	0.39399	C113	0.47626	1.07686	-0.599
C114	0.17037	0.44123	0.39344	C114	0.46193	1.06498	-0.81435
C115	0.18287	0.46282	0.32676	C115	0.43728	1.07782	-0.89298
C116	0.15687	0.48283	0.33318	C116	0.47456	1.015	0.58337
C117	0.11821	0.48185	0.40899	H117	0.39115	1.10743	-1.04753
C118	0.10514	0.46	0.4716	H118	0.43363	1.13728	-0.45329
C119	0.1309	0.43977	0.4631	H119	0.47805	1.11307	-0.30177
H120	0.21852	0.68203	0.26549	H120	0.47046	1.04456	-0.92725
H121	0.18277	0.64227	0.25402	H121	0.42539	1.06724	-1.06268
H122	0.27067	0.61553	0.56658	H122	0.45332	1.02673	0.65295
H123	0.30547	0.65564	0.58262	N123	0.44081	0.5	0.40258
H124	0.21294	0.46426	0.27113	C124	0.47083	0.5	0.44742
H125	0.16731	0.49946	0.28277	N125	0.5	0.06158	0.5
H126	0.07572	0.45899	0.53184	C126	0.5	0.03041	0.5
H127	0.12073	0.4232	0.51518	C127	0.03004	0	0.40285
H128	0.23157	0.42386	0.36409	N128	0.06066	0	0.30233
H129	-0.00181	0.21568	0.86099	N129	0	0.43849	0.5
H130	-0.01144	0.2338	0.70169	C130	0	0.46959	0.5
H131	0.4843	0.70481	0.65298				
H132	0.49595	0.72117	0.82853				
N133	0.32008	0.11085	0.51656				
C134	0.30359	0.08926	0.51783				
H135	0.27085	0.08678	0.54294				
C136	0.27679	0.31226	0.49524				
C137	0.31774	0.31001	0.44193				
C138	0.33569	0.28717	0.41721				
C139	0.25487	0.29132	0.54205				
C140	0.21619	0.29304	0.61755				
C141	0.19267	0.27236	0.64721				

C142	0.20727	0.24917	0.60449
C143	0.18275	0.22802	0.62545
C144	0.31179	0.26596	0.44409
C145	0.32628	0.24271	0.40116
C146	0.30283	0.22202	0.43104
C147	0.26417	0.22374	0.50638
C148	0.24102	0.20278	0.54448
C149	0.20017	0.20518	0.59711
C150	0.24775	0.24699	0.54105
C151	0.27168	0.2681	0.51047
H152	0.33521	0.32609	0.41157
H153	0.35555	0.24033	0.34118
H154	0.3149	0.2046	0.39177
H155	0.18179	0.18903	0.61897
C156	0.20658	0.75829	0.50844
C157	0.19211	0.73523	0.54818
C158	0.21735	0.71508	0.52977
C159	0.25784	0.71721	0.46952
C160	0.28248	0.69654	0.43893
C161	0.26386	0.78426	0.42117
C162	0.30449	0.78633	0.36188
C163	0.33003	0.76625	0.34698
C164	0.31551	0.74319	0.38666
C165	0.34109	0.7226	0.37181
C166	0.32415	0.69953	0.39676
C167	0.27392	0.74049	0.43815
C168	0.24808	0.76103	0.45577
H169	0.36108	0.76884	0.30157
H170	0.34314	0.68366	0.37588
C171	0.379	0.28604	0.35875
C172	0.25971	0.17841	0.54204
C173	0.40943	0.27478	0.44233
C174	0.45001	0.27299	0.38503
C175	0.46081	0.28292	0.24568
C176	0.43079	0.29462	0.16353
C177	0.39004	0.29615	0.21949
C178	0.29721	0.17528	0.61663

C179	0.31646	0.15284	0.6092
C180	0.29756	0.1329	0.53403
C181	0.25878	0.13548	0.46687
C182	0.24006	0.15822	0.46974
N183	0.50218	0.28101	0.18723
H184	0.40154	0.26738	0.5515
H185	0.47322	0.26412	0.44953
H186	0.43898	0.30226	0.05546
H187	0.36698	0.30491	0.15364
H188	0.31178	0.19042	0.67827
H189	0.3462	0.15081	0.66188
H190	0.24408	0.12023	0.40631
H191	0.21099	0.16024	0.41209
C192	0.38535	0.72435	0.32637
C193	0.4145	0.73447	0.42876
C194	0.45663	0.735	0.38846
C195	0.46994	0.72537	0.24619
C196	0.44088	0.71552	0.1434
C197	0.39878	0.71509	0.18283
N198	0.51296	0.72535	0.20669
H199	0.40463	0.74163	0.54006
H200	0.4791	0.74252	0.4684
H201	0.45095	0.708	0.03345
H202	0.37681	0.70754	0.10149
H203	0.20387	0.31052	0.65425
H204	0.16305	0.27468	0.70499
H205	0.16114	0.7326	0.59422
H206	0.20502	0.69775	0.56438
H207	0.31673	0.80354	0.32481
H208	0.52403	0.27077	0.24157
H209	0.51129	0.28971	0.08858
H210	0.52339	0.71737	0.10337
H211	0.53484	0.73335	0.2802
C212	0.75427	0.8102	0.40815
C213	0.7959	0.8105	0.35999
C214	0.81831	0.78901	0.33908
C215	0.73565	0.78802	0.45042

C216	0.69617	0.78727	0.51979
C217	0.79777	0.76646	0.36012
C218	0.81628	0.74433	0.31549
C219	0.75702	0.76604	0.42233
H220	0.81094	0.82767	0.33655
C221	0.73032	0.83297	0.40367
C222	0.86313	0.79073	0.29726
C223	0.89267	0.77824	0.37869
C224	0.93449	0.77891	0.33449
C225	0.94752	0.79254	0.21133
C226	0.91856	0.8058	0.13389
C227	0.87668	0.80505	0.17715
C228	0.74592	0.85487	0.46915
C229	0.72337	0.87622	0.46279
C230	0.68493	0.87598	0.39009
C231	0.66946	0.85427	0.32373
C232	0.69234	0.83302	0.32723
N233	0.99013	0.79301	0.16536
H234	0.88318	0.76777	0.47523
H235	0.95683	0.76885	0.39572
H236	0.92851	0.81656	0.03946
H237	0.85471	0.81526	0.11505
H238	0.77522	0.85537	0.52672
H239	0.73529	0.89277	0.51789
H240	0.63984	0.85405	0.26762
H241	0.68032	0.81651	0.27141
C242	0.13712	0.77707	0.57425
C243	0.12552	0.78546	0.72176
C244	0.08411	0.78399	0.7701
C245	0.05399	0.77402	0.67196
C246	0.06546	0.76568	0.52448
C247	0.10687	0.76723	0.47551
N248	0.01188	0.77222	0.72256
H249	0.14851	0.79279	0.79995
H250	0.07545	0.79035	0.88438
H251	0.04225	0.75801	0.44816
H252	0.11542	0.76086	0.36097

N253	0.66041	0.89736	0.38753
H254	0.67985	0.80377	0.55137
H255	0.84552	0.74386	0.25349
H256	1.00053	0.80415	0.07911
H257	1.01186	0.78282	0.22065
H258	-0.0108	0.76456	0.65131
H259	0.00307	0.77792	0.83322
C260	0.25574	0.82984	0.44271
C261	0.28996	0.83626	0.53285
C262	0.30727	0.85951	0.53048
C263	0.29063	0.87686	0.43921
C264	0.25484	0.87099	0.35472
C265	0.23763	0.8475	0.35594
H266	0.30352	0.82301	0.60319
H267	0.3343	0.86407	0.59791
H268	0.24023	0.88426	0.2876
H269	0.2107	0.84297	0.28767
C270	0.18086	0.7788	0.52272
C271	0.19711	0.80172	0.49116
C272	0.23852	0.80483	0.44574
H273	0.1777	0.8175	0.50894
N274	0.31208	0.89975	0.43394
C275	0.32975	0.06783	0.4926
C276	0.36826	0.06958	0.41674
C277	0.39225	0.04894	0.38882
C278	0.37853	0.02633	0.43895
C279	0.34037	0.02481	0.51794
C280	0.31594	0.04531	0.54275
C281	0.44706	1.00535	0.40912
N282	0.40239	1.00478	0.40537
C283	0.51133	0.0195	0.52678
C284	0.51347	0.99119	0.30826
C285	0.46802	0.01947	0.52144
C286	0.47018	0.99122	0.30261
H287	0.37925	0.08688	0.37661
H288	0.42122	0.05042	0.32613
H289	0.32944	0.00763	0.55946

H290	0.28646	0.04366	0.60238
H291	0.52704	0.03031	0.61478
H292	0.53087	0.98036	0.22459
H293	0.45067	0.03015	0.60601
H294	0.45449	0.98032	0.21494
C295	0.30544	0.91734	0.34431
C296	0.33211	0.93929	0.35339
C297	0.324	0.95885	0.26195
C298	0.34825	0.98008	0.2747
C299	0.38085	0.98199	0.37859
C300	0.38981	0.96221	0.46625
C301	0.36563	0.94101	0.45404
H302	0.29848	0.95786	0.18313
H303	0.34121	0.99536	0.2068
H304	0.41464	0.96365	0.54772
H305	0.3726	0.92608	0.52507
H306	0.2811	0.91615	0.2611
C307	0.65185	0.06969	0.48466
C308	0.62003	0.06813	0.3768
C309	0.59497	0.04724	0.36227
C310	0.6015	0.02761	0.4544
C311	0.63246	0.02939	0.56481
C312	0.65744	0.0503	0.5802
C313	0.53438	0.00524	0.42086
N314	0.57896	0.00513	0.4276
H315	0.61504	0.08295	0.30255
H316	0.57134	0.04592	0.27607
H317	0.63757	0.01427	0.63647
H318	0.68157	0.05116	0.66468
C319	0.67587	0.91894	0.36896
C320	0.6493	0.94044	0.37581
C321	0.66446	0.96219	0.31804
C322	0.64071	0.98313	0.33049
C323	0.60173	0.9829	0.40399
C324	0.5863	0.96099	0.45979
C325	0.60975	0.93985	0.44475
H326	0.69477	0.96299	0.26369

H327	0.65294	0.99974	0.28473
H328	0.55657	0.96047	0.5182
H329	0.59768	0.92319	0.49066
H330	0.70867	0.92141	0.34495
C331	0.1768	0.28404	2.0957
C332	0.19253	0.30687	2.05812
C333	0.23385	0.30993	2.0115
C334	0.20303	0.2637	2.08659
C335	0.18939	0.24088	2.13417
C336	0.21517	0.22088	2.12063
C337	0.25524	0.22286	2.05652
C338	0.28033	0.20223	2.02983
C339	0.25936	0.28941	1.9897
C340	0.29963	0.29142	1.92703
C341	0.32541	0.27145	1.91394
C342	0.31158	0.24861	1.96003
C343	0.33747	0.22819	1.94741
C344	0.32156	0.20532	1.98205
C345	0.27047	0.24597	2.01684
C346	0.24425	0.26637	2.03094
H347	0.1729	0.3226	2.07224
H348	0.1587	0.23836	2.1831
H349	0.20357	0.20376	2.16153
H350	0.31135	0.30848	1.88572
H351	0.35622	0.27398	1.86605
H352	0.34081	0.18954	1.96178
C353	0.19282	0.7662	2.0498
C354	0.20823	0.74324	2.00485
C355	0.18423	0.72177	2.02165
C356	0.30607	0.71751	1.84159
C357	0.26654	0.71863	1.91077
C358	0.24394	0.69728	1.94561
C359	0.20245	0.69912	1.99273
C360	0.24918	0.74165	1.94562
H361	0.16265	0.76807	2.10408
H362	0.31897	0.70032	1.80182
H363	0.18457	0.68277	2.01401

N364	0.21169	0.10571	1.98655
C365	0.18766	0.09718	1.87859
H366	0.1814	0.10776	1.78077
C367	0.13281	0.28213	2.14423
C368	0.25137	0.33484	2.00558
C369	0.26242	0.1773	2.02401
C370	0.38056	0.22973	1.88916
C371	0.28511	0.34132	2.09792
C372	0.30452	0.36389	2.08748
C373	0.29072	0.3804	1.98463
C374	0.25514	0.37469	1.89844
C375	0.23557	0.3519	1.90885
C376	0.11963	0.29175	2.28728
C377	0.07777	0.29059	2.33002
C378	0.04877	0.2797	2.23089
C379	0.06192	0.26962	2.0896
C380	0.10372	0.27101	2.04569
C381	0.41288	0.23635	1.9881
C382	0.45385	0.23685	1.93334
C383	0.46277	0.23063	1.78013
C384	0.43048	0.22426	1.68111
C385	0.38952	0.22389	1.73516
C386	0.2275	0.17233	1.93454
C387	0.21067	0.14897	1.92099
C388	0.22927	0.12981	1.99342
C389	0.26445	0.13464	2.0817
C390	0.28113	0.15826	2.0969
N391	0.3319	0.60813	1.95792
C392	0.31735	0.58625	1.97125
C393	0.34511	0.56527	1.95861
N394	0.00597	0.27897	2.27328
N395	0.50458	0.23068	1.72526
C396	0.38381	0.56665	1.88339
C397	0.40846	0.546	1.86406
C398	0.39509	0.52383	1.92185
C399	0.35712	0.52289	2.0026
C400	0.33216	0.54329	2.01904

C401	0.46288	0.50151	1.89379
N402	0.41819	0.50188	1.89037
C403	0.52801	0.51437	2.01038
C404	0.52814	0.48425	1.80157
C405	0.48483	0.51586	2.00217
C406	0.48497	0.48573	1.79343
H407	0.29704	0.32846	2.17467
H408	0.33137	0.36836	2.15604
H409	0.24267	0.3875	1.82263
H410	0.20899	0.34736	1.83886
H411	0.14155	0.3	2.36621
H412	0.06784	0.29817	2.44009
H413	0.03962	0.26082	2.01407
H414	0.11347	0.26363	1.93481
H415	0.40635	0.24093	2.10727
H416	0.47879	0.24181	2.00999
H417	0.43716	0.21957	1.56236
H418	0.36485	0.21908	1.65726
H419	0.21323	0.1867	1.87536
H420	0.18272	0.14619	1.85623
H421	0.27878	0.11998	2.13845
H422	0.30826	0.16172	2.16605
H423	0.39423	0.58355	1.8359
H424	0.43734	0.54705	1.80039
H425	0.34645	0.5061	2.05033
H426	0.30267	0.5419	2.07881
H427	0.54434	0.52536	2.09592
H428	0.54462	0.47216	1.72224
H429	0.46832	0.52779	2.08234
H430	0.46856	0.47457	1.70887
H431	0.28482	0.58322	1.99787
N432	0.31507	0.4021	1.96856
C433	0.31258	0.41783	1.86346
C434	0.34198	0.43857	1.86238
C435	0.33703	0.45691	1.75869
C436	0.36353	0.47725	1.76283
C437	0.39539	0.47939	1.86965

C438	0.40146	0.46064	1.9688
C439	0.37491	0.44038	1.96565
H440	0.31204	0.4558	1.67766
H441	0.35864	0.49181	1.68666
H442	0.42564	0.46234	2.05274
H443	0.37949	0.42633	2.0459
H444	0.28991	0.41581	1.77442
C445	0.14021	0.72232	2.07129
C446	0.26461	0.67347	1.95343
C447	0.30191	0.67227	2.03236
C448	0.3236	0.65065	2.03385
C449	0.30717	0.62948	1.96464
C450	0.26831	0.63003	1.89489
C451	0.24727	0.65201	1.88793
C452	0.1107	0.73301	1.98085
C453	0.06917	0.73388	2.02852
C454	0.05667	0.72371	2.16529
C455	0.08586	0.71252	2.254
C456	0.12751	0.7119	2.20771
N457	0.70018	0.59791	1.91702
C458	0.70068	0.58043	2.0115
C459	0.67185	0.55952	1.99179
N460	0.01436	0.72471	2.21405
C461	0.64111	0.55905	1.87973
C462	0.61472	0.53884	1.85966
C463	0.61881	0.5188	1.9504
C464	0.64884	0.51943	2.06434
C465	0.67512	0.53968	2.08536
C466	0.55006	0.49843	1.91081
N467	0.59461	0.49704	1.91934
H468	0.31439	0.68835	2.09051
H469	0.35321	0.65022	2.08957
H470	0.25522	0.61373	1.84082
H471	0.21822	0.65241	1.82815
H472	0.11998	0.74055	1.87349
H473	0.04652	0.74215	1.95841
H474	0.07634	0.70452	2.35948

H475	0.1499	0.70357	2.27869
H476	0.63791	0.57424	1.80681
H477	0.59187	0.53837	1.77065
H478	0.65213	0.50395	2.13465
H479	0.69845	0.53969	2.1727
H480	0.72145	0.58102	2.10708
C481	0.76856	0.66439	1.93189
C482	0.73557	0.66023	1.8317
C483	0.7141	0.6381	1.82697
C484	0.72524	0.61964	1.92127
C485	0.75978	0.62319	2.01625
C486	0.78122	0.64553	2.02177
N487	0.67195	0.38818	1.89281
C488	0.68802	0.40934	1.86589
C489	0.66221	0.43123	1.8711
C490	0.67791	0.45259	1.80976
C491	0.65488	0.47385	1.82026
C492	0.61602	0.47434	1.8948
C493	0.59991	0.4528	1.9534
C494	0.62269	0.43135	1.94078
H495	0.72618	0.67439	1.759
H496	0.68812	0.63532	1.75145
H497	0.77041	0.60893	2.08551
H498	0.80722	0.64825	2.09755
H499	0.70814	0.45287	1.75448
H500	0.66766	0.49019	1.77274
H501	0.57029	0.45286	2.01267
H502	0.6101	0.415	1.98904
H503	0.72054	0.4111	1.83494
H504	0.51158	0.22662	1.61058
H505	0.52893	0.23533	1.79826
H506	-0.01596	0.27114	2.19933
H507	-0.00441	0.28643	2.3779
H508	0.0041	0.71544	2.30896
H509	-0.00728	0.73412	2.15295
C510	0.81893	0.07708	1.91122
H511	0.78623	0.07254	1.92053

C512	0.72462	0.75458	1.98057
C513	0.70517	0.7323	2.012
C514	0.7269	0.71074	2.00202
C515	0.76875	0.71038	1.95619
C516	0.79038	0.68828	1.93671
C517	0.78826	0.77703	1.91259
C518	0.8312	0.7768	1.88323
C519	0.85288	0.75523	1.87554
C520	0.83247	0.73286	1.89442
C521	0.85408	0.71073	1.88095
C522	0.83288	0.68883	1.90358
C523	0.78957	0.73267	1.93061
C524	0.76742	0.75478	1.94092
H525	0.88602	0.75629	1.85585
H526	0.8492	0.67187	1.88577
C527	0.89812	0.70964	1.83236
C528	0.9279	0.70096	1.93015
C529	0.96958	0.70017	1.88417
C530	0.98182	0.70805	1.74063
C531	0.95205	0.71642	1.64217
C532	0.91034	0.71706	1.68714
N533	1.02443	0.70764	1.6951
H534	0.91886	0.69508	2.04223
H535	0.99251	0.69367	1.96039
H536	0.96122	0.72249	1.53108
H537	0.88757	0.72351	1.60973
H538	0.6728	0.73111	2.04372
H539	0.71053	0.69428	2.03171
H540	0.84892	0.7933	1.86813
H541	1.03423	0.71507	1.59317
H542	1.04675	0.70078	1.76568
C543	0.27856	0.80748	1.92396
C544	0.32004	0.80551	1.8764
C545	0.33822	0.78285	1.84806
C546	0.25577	0.78612	1.95824
C547	0.21631	0.78721	2.0282
C548	0.3141	0.76144	1.86449

C549	0.32957	0.73848	1.82034
C550	0.27307	0.76309	1.92302
H551	0.33809	0.82179	1.85508
C552	0.25851	0.83155	1.9186
C553	0.38238	0.78221	1.79988
C554	0.41159	0.77116	1.8901
C555	0.45327	0.77051	1.84431
C556	0.4662	0.781	1.70884
C557	0.43725	0.79228	1.61966
C558	0.39551	0.79292	1.66483
C559	0.27757	0.85286	1.97954
C560	0.25824	0.87533	1.96963
C561	0.21928	0.87661	1.90146
C562	0.20086	0.85553	1.83836
C563	0.22076	0.83335	1.84316
N564	0.50858	0.77994	1.66084
H565	0.40199	0.76306	1.99562
H566	0.47562	0.76199	1.91436
H567	0.44709	0.80033	1.51459
H568	0.37335	0.80146	1.59387
H569	0.30689	0.85203	2.03716
H570	0.27288	0.89145	2.01996
H571	0.17113	0.8565	1.78401
H572	0.20668	0.81742	1.78865
C573	0.65776	0.77815	2.02756
C574	0.6428	0.77023	2.16821
C575	0.60098	0.77267	2.209
C576	0.57371	0.78337	2.11045
C577	0.58854	0.79165	1.97111
C578	0.63032	0.78912	1.92984
N579	0.53109	0.78606	2.15224
H580	0.66373	0.76225	2.24592
H581	0.58987	0.76651	2.31785
H582	0.56765	0.80018	1.89539
H583	0.64136	0.79581	1.82217
N584	0.19637	0.89865	1.90229
H585	0.20349	0.80438	2.06848

H586	0.35982	0.73654	1.76709
H587	0.51896	0.78856	1.56344
H588	0.52971	0.7699	1.71995
H589	0.51098	0.79507	2.08279
H590	0.51906	0.77873	2.2516
C591	0.78582	0.82311	1.90191
C592	0.78228	0.84086	2.02061
C593	0.80286	0.86319	2.01143
C594	0.82616	0.86851	1.88162
C595	0.82906	0.8511	1.76019
C596	0.80853	0.8286	1.76969
H597	0.76406	0.83722	2.12133
H598	0.80057	0.87657	2.10508
H599	0.84775	0.85469	1.66015
H600	0.81102	0.81522	1.67598
C601	0.70253	0.77663	1.98676
C602	0.72343	0.79829	1.9548
C603	0.76581	0.79893	1.91955
H604	0.70653	0.81516	1.96096
N605	0.84771	0.8914	1.88023
C606	0.84895	0.05709	1.89635
C607	0.8898	0.06099	1.844
C608	0.91721	0.04154	1.82548
C609	0.90463	0.01794	1.86222
C610	0.86383	0.01431	1.91677
C611	0.83625	0.03358	1.93209
C612	0.97675	0.99998	1.85191
N613	0.93228	0.99757	1.84007
C614	0.03795	0.01627	1.98139
C615	0.04571	0.98685	1.7706
C616	0.9948	0.0151	1.96623
C617	1.00266	0.98585	1.75444
H618	0.90006	0.07903	1.81464
H619	0.94805	0.04482	1.78037
H620	0.85321	-0.00356	1.94738
H621	0.80499	0.03015	1.97317
H622	0.05157	0.02809	2.06943

H623	0.06525	0.9761	1.6935
H624	0.97538	0.02567	2.04472
H625	0.98932	0.97405	1.6658
C626	0.85627	0.90357	1.76008
C627	0.87779	0.92731	1.7768
C628	0.87433	0.94435	1.6636
C629	0.89252	0.96748	1.68396
C630	0.91502	0.97356	1.81573
C631	0.91959	0.95637	1.92649
C632	0.90083	0.93344	1.90796
H633	0.85666	0.93998	1.56186
H634	0.88903	0.98082	1.59839
H635	0.93692	0.96109	2.02841
H636	0.90399	0.92048	1.99616
H637	0.84522	0.89733	1.6489
C638	0.16744	0.07314	1.88632
C639	0.1389	0.06577	1.77384
C640	0.11867	0.04324	1.77976
C641	0.1269	0.02774	1.89728
C642	0.15516	0.03508	2.01043
C643	0.17531	0.05769	2.00535
C644	0.06366	0.00201	1.88445
N645	0.10805	0.00395	1.89688
H646	0.13223	0.07745	1.68153
H647	0.09711	0.03755	1.69151
H648	0.16158	0.02311	2.10134
H649	0.1971	0.06315	2.09349
C650	0.21232	0.92033	1.89434
C651	0.18521	0.94163	1.89554
C652	0.20034	0.96348	1.83927
C653	0.17482	0.9837	1.8372
C654	0.13389	0.9827	1.89483
C655	0.11897	0.96079	1.95237
C656	0.14424	0.94038	1.95149
H657	0.23171	0.96478	1.79444
H658	0.18705	1.00017	1.78978
H659	0.08785	0.9596	1.99906

H660	0.13208	0.92363	1.99595
H661	0.2454	0.92291	1.87772
C662	0.67679	1.2535	1.03218
C663	0.69513	1.23117	0.9904
C664	0.67317	1.20885	1.00187
C665	0.79668	1.20937	0.83565
C666	0.75662	1.20887	0.90077
C667	0.73584	1.18662	0.92973
C668	0.69372	1.18694	0.97052
C669	0.73658	1.23117	0.9352
H670	0.6455	1.25441	1.07733
H671	0.81177	1.19278	0.79695
H672	0.67739	1.16996	0.98922
N673	0.83142	1.09961	0.92101
C674	0.62923	1.20765	1.0529
C675	0.75842	1.16326	0.9338
C676	0.79537	1.16235	1.01544
C677	0.81883	1.14127	1.01123
C678	0.80477	1.12025	0.93254
C679	0.7663	1.12045	0.8595
C680	0.74329	1.14186	0.86004
C681	0.59859	1.1989	0.95214
C682	0.55704	1.19795	1.00025
C683	0.54581	1.20569	1.14913
C684	0.57637	1.21425	1.25009
C685	0.61789	1.21507	1.20258
N686	0.50345	1.2048	1.19783
H687	0.80631	1.17823	1.07993
H688	0.84811	1.14116	1.06943
H689	0.75493	1.1044	0.7974
H690	0.7144	1.14194	0.79909
H691	0.60687	1.19306	0.83629
H692	0.53346	1.19138	0.92177
H693	0.56793	1.22026	1.36526
H694	0.64122	1.22182	1.28116
H695	0.49464	1.21094	1.30761
H696	0.48058	1.19767	1.1251

C697	0.75845	1.29811	0.91596
C698	0.80014	1.29766	0.86953
C699	0.82107	1.27582	0.84291
C700	0.73806	1.27586	0.94885
C701	0.69835	1.27531	1.01645
C702	0.79946	1.25351	0.85827
C703	0.81778	1.23124	0.81566
C704	0.75808	1.25354	0.9143
H705	0.81667	1.31455	0.85116
C706	0.73634	1.32172	0.91427
C707	0.8658	1.27723	0.80291
C708	0.89504	1.2668	0.89596
C709	0.9374	1.26961	0.86458
C710	0.95101	1.28316	0.74174
C711	0.92199	1.29341	0.648
C712	0.87958	1.29048	0.67824
C713	0.75516	1.34359	0.96814
C714	0.73486	1.36571	0.9569
C715	0.695	1.36622	0.89533
C716	0.67629	1.34459	0.8421
C717	0.69721	1.32272	0.84735
N718	0.99425	1.28689	0.71391
H719	0.88497	1.25692	0.9943
H720	0.95972	1.26162	0.93711
H721	0.93233	1.30381	0.5525
H722	0.85749	1.29863	0.60524
H723	0.78529	1.34354	1.02052
H724	0.74963	1.3823	1.00012
H725	0.6457	1.34489	0.79367
H726	0.68284	1.30644	0.79896
H727	0.68347	1.29184	1.05759
H728	0.8484	1.23056	0.76403
H729	1.00474	1.29934	0.6354
H730	1.01617	1.27876	0.7804
N731	0.67612	1.10875	0.40484
C732	0.74167	1.17624	0.41716
C733	0.70488	1.17277	0.33564

C734	0.68426	1.15035	0.33081
C735	0.70015	1.13086	0.40583
C736	0.73846	1.13369	0.47935
C737	0.75891	1.15634	0.48621
H738	0.69178	1.18768	0.27816
H739	0.6553	1.14816	0.27101
H740	0.75263	1.11855	0.53097
H741	0.78778	1.15842	0.54696
C742	0.69392	1.26527	0.49735
C743	0.67691	1.24263	0.5347
C744	0.7001	1.22162	0.51846
C745	0.74044	1.22214	0.456
C746	0.76252	1.2005	0.42249
C747	0.75432	1.28909	0.41309
C748	0.7951	1.28969	0.35437
C749	0.81797	1.26859	0.33544
C750	0.80074	1.24602	0.37265
C751	0.82376	1.22445	0.35437
C752	0.80422	1.20203	0.37728
C753	0.75907	1.24486	0.42545
C754	0.73576	1.26643	0.44565
H755	0.84908	1.27004	0.28863
H756	0.82154	1.18552	0.35597
C757	0.86839	1.22457	0.31286
C758	0.8974	1.23517	0.414
C759	0.93976	1.23401	0.37932
C760	0.95356	1.22191	0.24493
C761	0.92469	1.21171	0.14255
C762	0.88228	1.21312	0.17588
N763	0.99685	1.21981	0.21326
H764	0.88716	1.244	0.52028
H765	0.96198	1.24226	0.45733
H766	0.93514	1.20261	0.03756
H767	0.86035	1.20535	0.09507
H768	0.64524	1.24073	0.57447
H769	0.6859	1.20482	0.55399
H770	0.80945	1.30652	0.31992

H771	1.0077	1.20894	0.12148
H772	1.01858	1.22823	0.28586
C773	0.62609	1.28718	0.55872
C774	0.61392	1.27938	0.70333
C775	0.57272	1.28134	0.75325
C776	0.54325	1.29134	0.66028
C777	0.55524	1.29937	0.51695
C778	0.59638	1.29728	0.46609
N779	0.50126	1.29346	0.71164
H780	0.63639	1.27191	0.77762
H781	0.56374	1.27533	0.86488
H782	0.53258	1.30726	0.44521
H783	0.60519	1.30365	0.35493
H784	0.47962	1.30235	0.64789
H785	0.49106	1.28528	0.81078
C786	0.75175	1.33494	0.43861
C787	0.78563	1.33987	0.53367
C788	0.80651	1.36219	0.53122
C789	0.7938	1.38006	0.43513
C790	0.75816	1.3758	0.34674
C791	0.73732	1.35329	0.34837
H792	0.79623	1.32617	0.60746
H793	0.83329	1.36554	0.60209
H794	0.74627	1.38967	0.27717
H795	0.7105	1.34999	0.27728
C796	0.67028	1.28676	0.50852
C797	0.68946	1.30905	0.47759
C798	0.73146	1.31061	0.43773
H799	0.67207	1.32563	0.49507
N800	0.81829	1.40199	0.43125

S21. References

[1] V. Khomenko, E. Frackowiak, F. Béguin, *Electrochim. Acta* **2005**, *50*, 2499.

[2] J. W. Lee, A. S. Hall, J.-D. Kim, T. E. Mallouk, *Chem. Mater.* **2012**, *24*, 1158.

[3] S. Jin, T. Sakurai, T. Kowalczyk, S. Dalapati, F. Xu, H. Wei, X. Chen, J. Gao, S. Seki, S. Irle, D. Jiang, *Chemistry* **2014**, *20*, 14608.

[4] T. Ma, E. A. Kapustin, S. X. Yin, L. Liang, Z. Zhou, J. Niu, L.-H. Li, Y. Wang, J. Su, J. Li, X. Wang, W. D. Wang, W. Wang, J. Sun, O. M. Yaghi, *Science* **2018**, *361*, 48.

[5] A. F. M. El-Mahdy, M. B. Zakaria, H.-X. Wang, T. Chen, Y. Yamauchi, S.-W. Kuo, *J. Mater. Chem. A* **2020**, *8*, 25148.

[6] A. F. M. El-Mahdy, C. Young, J. Kim, J. You, Y. Yamauchi, S.-W. Kuo, *ACS Appl. Mater. Interfaces* **2019**, *11*, 9343.

[7] X. Zhao, M. Sajjad, Y. Zheng, M. Zhao, Z. Li, Z. Wu, K. Kang, L. Qiu, *Carbon* **2021**, *182*, 144.

[8] C. R. DeBlase, K. E. Silberstein, T.-T. Truong, H. D. Abruña, W. R. Dichtel, *J. Am. Chem. Soc.* **2013**, *135*, 16821.

[9] Y. Dong, Y. Wang, X. Zhang, Q. Lai, Y. Yang, J. *Chem. Eng.* **2022**, *449*, 137858.

[10] J. Zhang, Y. Wang, T. Yang, S. Liu, J. Li, J. Fan, Z. Wu, L. Qiu, *J. Colloid Interface Sci.* **2024**, *677*, 1037.

[11] A. Khayum M, V. Vijayakumar, S. Karak, S. Kandambeth, M. Bhadra, K. Suresh, N. Acharambath, S. Kurungot, R. Banerjee, *ACS Appl. Mater. Interfaces* **2018**, *10*, 28139.

[12] S. Kandambeth, J. Jia, H. Wu, V. S. Kale, P. T. Parvatkar, J. Czaban-Jóźwiak, S. Zhou, X. Xu, Z. O. Ameur, E. Abou-Hamad, A.-H. Emwas, O. Shekhah, H. N. Alshareef, M. Eddaoudi, *Adv. Energy Mater.* **2020**, *10*, 38.

[13] S. Haldar, R. Kushwaha, R. Maity, R. Vaidhyanathan, *ACS Mater. Lett.* **2019**, *1*, 490.

[14] L. Li, F. Lu, R. Xue, B. Ma, Q. Li, N. Wu, H. Liu, W. Yao, H. Guo, W. Yang, *ACS Appl. Mater. Interfaces* **2019**, *11*, 26355.

[15] M. R. Biradar, C. R. K. Rao, S. V. Bhosale, S. V. Bhosale, *Energy Fuels* **2023**, *37*, 4671.

[16] M. Sajjad, R. Tao, L. Qiu, *J. Mater. Sci: Mater. Electron* **2021**, *32*, 1602.

[17] K. Kang, Z. Wu, M. Zhao, Z. Li, Y. Ma, J. Zhang, Y. Wang, M. Sajjad, R. Tao, L. Qiu, *ChemComm* **2022**, *58*, 3649.

[18] A. Khojastehnezhad, K. Rhili, M. K. Shehab, H. Gamraoui, Z. Peng, A. Samih ElDouhaibi, R. Touzani, B. Hammouti, H. M. El-Kaderi, M. Siaj, *ACS Appl. Energy Mater.* **2023**, *6*, 12216.

[19] A. M. Khattak, Z. A. Ghazi, B. Liang, N. A. Khan, A. Iqbal, L. Li, Z. Tang, *J. Mater. Chem. A* **2016**, *4*, 16312.

[20] S. K. Das, K. Bhunia, A. Mallick, A. Pradhan, D. Pradhan, A. Bhaumik, *Microporous Mesoporous Mater.* **2018**, *266*, 109.

[21] A. F. M. El-Mahdy, C.-H. Kuo, A. Alshehri, C. Young, Y. Yamauchi, J. Kim, S.-W. Kuo, *J. Mater. Chem. A* **2018**, *6*, 19532.

[22] A. Halder, M. Ghosh, A. Khayum M, S. Bera, M. Addicoat, H. S. Sasmal, S. Karak, S. Kurungot, R. Banerjee, *J. Am. Chem. Soc.* **2018**, *140*, 10941.

[23] S. Chandra, D. Roy Chowdhury, M. Addicoat, T. Heine, A. Paul, R. Banerjee, *Chem. Mater.* **2017**, *29*, 2074.

[24] A. F. M. El-Mahdy, M. G. Mohamed, T. H. Mansoure, H.-H. Yu, T. Chen, S.-W. Kuo, *ChemComm* **2019**, *55*, 14890.

[25] A. F. M. El-Mahdy, Y.-H. Hung, T. H. Mansoure, H.-H. Yu, T. Chen, S.-W. Kuo, *Chem. Asian J.* **2019**, *14*, 1429.

[26] S. Li, B. Kumbhakar, B. Mishra, J. Roeser, N. Chaoui, J. Schmidt, A. Thomas, P. Pachfule, *ACS Appl. Energy Mater.* **2023**, *6*, 9256.

[27] R. Iqbal, M. K. Majeed, A. Hussain, A. Ahmad, M. Ahmad, B. Jabar, A. R. Akbar, S. Ali, S. Rauf, A. Saleem, *Mater. Chem. Front.* **2023**, *7*, 2464.

[28] M. Li, J. Liu, Y. Li, G. Xing, X. Yu, C. Peng, L. Chen, *CCS Chemistry* **2021**, 3, 696-706.
